# The Analysis of Mutant Alleles of Different Strength Reveals Multiple Functions of Topoisomerase 2 in Regulation of *Drosophila* Chromosome Structure

**DOI:** 10.1371/journal.pgen.1004739

**Published:** 2014-10-23

**Authors:** Valentina Mengoli, Elisabetta Bucciarelli, Ramona Lattao, Roberto Piergentili, Maurizio Gatti, Silvia Bonaccorsi

**Affiliations:** 1 Istituto Pasteur-Fondazione Cenci Bolognetti and Istituto di Biologia e Patologia Molecolari (IBPM) del CNR, Dipartimento di Biologia e Biotecnologie “C. Darwin”, Sapienza, Università di Roma, Roma, Italy; 2 Institute of Molecular and Cellular Biology SB RAS, Novosibirsk, Russia; The University of North Carolina at Chapel Hill, United States of America

## Abstract

Topoisomerase II is a major component of mitotic chromosomes but its role in the assembly and structural maintenance of chromosomes is rather controversial, as different chromosomal phenotypes have been observed in various organisms and in different studies on the same organism. In contrast to vertebrates that harbor two partially redundant Topo II isoforms, *Drosophila* and yeasts have a single Topo II enzyme. In addition, fly chromosomes, unlike those of yeast, are morphologically comparable to vertebrate chromosomes. Thus, *Drosophila* is a highly suitable system to address the role of Topo II in the assembly and structural maintenance of chromosomes. Here we show that modulation of Top2 function in living flies by means of mutant alleles of different strength and in vivo RNAi results in multiple cytological phenotypes. In weak *Top2* mutants, meiotic chromosomes of males exhibit strong morphological abnormalities and dramatic segregation defects, while mitotic chromosomes of larval brain cells are not affected. In mutants of moderate strength, mitotic chromosome organization is normal, but anaphases display frequent chromatin bridges that result in chromosome breaks and rearrangements involving specific regions of the Y chromosome and 3L heterochromatin. Severe Top2 depletion resulted in many aneuploid and polyploid mitotic metaphases with poorly condensed heterochromatin and broken chromosomes. Finally, in the almost complete absence of Top2, mitosis in larval brains was virtually suppressed and in the rare mitotic figures observed chromosome morphology was disrupted. These results indicate that different residual levels of Top2 in mutant cells can result in different chromosomal phenotypes, and that the effect of a strong Top2 depletion can mask the effects of milder Top2 reductions. Thus, our results suggest that the previously observed discrepancies in the chromosomal phenotypes elicited by Topo II downregulation in vertebrates might depend on slight differences in Topo II concentration and/or activity.

## Author Summary

Type II topoisomerases (Topo II) are enzymes that disentangle DNA molecules during essential cellular processes such as DNA replication, chromosome condensation and mitotic cell division. Topo II is a major component of mitotic chromosomes and it is a well known target for cancer chemotherapy. Topo II inhibitors block the Topo II enzymatic activity leading to extensive DNA damage, which ultimately kills the cancer cell. Thus, investigating the role of Topo II in the assembly and structural maintenance of chromosomes is not only relevant to understand chromosome biology but might also have a translational impact on cancer therapy. Here we used *Drosophila* as model system to analyze the effect of Topo II depletion on chromosome stability. We show that the chromosomal phenotypes of mutant flies vary with the amount of residual Topo II, ranging from site-specific chromosome breaks, variations in chromosome number (aneuploidy and poliploidy) and dramatic defects in chromosome morphology. The chromosomal phenotypes observed in flies recapitulate all phenotypes seen in Topo II-depleted vertebrate chromosomes, reconciling the phenotypic discrepancies reported in previous studies. In addition, our finding that the Topo II dependent phenotypes vary with the residual amount of the enzyme provides useful information on the possible outcome of cancer therapy with Topo II inhibitors.

## Introduction

Type II topoisomerases are large ATP-dependent homodimeric enzymes that transiently cleave double stranded DNA, pass a second DNA double helix through the break, and then reseal the break [1], [2]. In this way, Topo II enzymes solve a variety of topological problems that normally arise in double stranded DNA during processes such as replication, transcription, recombination and sister chromatid segregation [1], [2]. Topo II enzymes are structurally and functionally conserved, and the genomes of all eukaryotes harbor at least one Topo II enzyme. Vertebrates have two Topo II isoforms, alpha and beta [3]; these enzymes have identical catalytic activities but distinct localization patterns during mitosis. The beta isoform is primarily cytoplasmic, while most of Topo II alpha is concentrated in mitotic chromosomes [4]. In contrast, yeast and *Drosophila* have a single *Topoisomerase II* (*Top2*) gene. Notably, both the *Drosophila Top2* and each of the human *Topo II* genes can rescue the phenotype of yeast *Top2* mutants, highlighting the strong functional conservation of type II topoisomerases [5]–[7].

Topo II alpha is a major component of vertebrate mitotic chromosomes [8]–[10]. In vivo studies have shown that Topo II alpha has a dynamic behavior and that chromosome-associated Topo II alpha is rapidly exchanged with the cytoplasmic pool [4], [11], [12]. In fixed mitotic chromosomes, Topo II alpha exhibits a discontinuous localization pattern with Topo II alpha alternating with cohesin along chromatid axes [13], [14]. There is also evidence that in some systems Topo II alpha accumulates at centromeres in prometaphase and metaphase, suggesting a role of this enzyme in the regulation of centromere structure and/or cohesion [4], [12], [15]–[17].

Studies in yeast have shown that Top2 is not required for completion of DNA synthesis but plays essential roles in mitotic chromosome condensation and sister chromatid segregation. Failure to decatenate sister chromatids results in anaphase chromatin bridges that cause chromosome breakage during anaphase or cytokinesis [18]–[22]. Loss of Topo II activity does not affect S phase progression and disrupts sister chromatid separation also in vertebrate cells [2], [23]–[25]. However, the role of Topo II in vertebrate chromosome structure is rather controversial, possibly due to species-specific differences in chromosome organization and/or the different methods used to inhibit Topo II function (chemical inhibitors, immunodepletion, mutations or RNAi). For example, treatment of Indian muntjac cells with the Topo II inhibitor ICRF-193 caused frequent failures in sister chromatid individualization, the process by which duplicated DNA is resolved into two distinct chromatids [26]. In contrast, Topo II inhibition with ICRF-193 did not affect sister chromatid resolution in both Chinese hamster ovary cells (CHO) and baby hamster kidney (BHK) cells [27]. Moreover, Topo II alpha inhibition in different vertebrate systems resulted in a variety of chromosome morphologies ranging from relatively mild effects on the axial compaction of chromosomes to severe defects in chromosome condensation [14], [24], [28]–[32].

Although Topo II beta is not normally able to compensate for Topo II alpha loss, overexpression of Topo II beta in human cells can correct the defects caused by Topo II alpha depletion [33]. In addition, it has been shown that DT40 avian cells and human cells depleted of both Topo II alpha and Topo II beta display chromosomal defects more severe than those observed in cells lacking Topo II alpha alone [17], [24], [25], [32]. These results suggest that the two Topo II isoforms are partially redundant. Thus, studies of cells depleted of both Topo II alpha and Topo II beta are particularly relevant to define the role of Topo II in the maintenance of proper chromosomal architecture. An analysis of DT40 avian cells conditionally depleted of both Topo II isoforms showed that they exhibit extensive anaphase chromatin bridges, defective cytokinesis and polyploid cells [24]. Similar defects were observed in human cells lacking both Topo II alpha and Topo II beta [17], [25], [32]. In addition, cytological analysis showed that the latter cells exhibit severe defects in chromosome structure ranging from chromosome entangling to disrupted chromosome morphology [25].

Another controversial issue is the existence of a decatenation checkpoint triggered by loss of Topo II activity. The existence of such a checkpoint was suggested by studies in human cells showing that catalytic inhibitors of Topo II such as ICRF-187 or ICFR-193 are able to induce a caffeine-sensitive G2 delay that is dependent on ATR and BRCA1, but apparently independent of the DNA damage checkpoint [34]–[36]. However, a decatenation checkpoint is not present in both *S. cerevisiae* and *S. pombe*, in which *Top 2* mutations cause minimal cell cycle delays [2], . Recent RNAi-based studies have also shown that depletion of Topo II alpha alone or both Topo II alpha and II beta does not trigger a decatenation checkpoint in vertebrate cells [24], [25]. In addition, no G2 delay was observed in Topo II-depleted vertebrate cells that were also treated with ICRF-193 [24], [38]. In contrast, expression of certain mutant forms of Top2 resulted in a G2 arrest in budding yeast cells [22], [37]. Collectively, these results indicate that loss of Topo II, and thus DNA catenation per se, is not able to induce a cell cycle delay and that a G2 checkpoint is instead activated by specific DNA lesions caused by catalytically inactive forms of Topo II.

Several studies have addressed the role of Top2 in *Drosophila*. Early work showed that injection of anti-Top2 antibodies or Top2 inhibitors into live *Drosophila* embryos result in strong defects in chromosome condensation and sister chromatid segregation at anaphase [39]. Two RNAi-based studies on S2 tissue culture cells showed that Top2-depleted fixed cells exhibit defects in longitudinal compaction of chromosomes, defective sister chromatid segregation and extensive anaphase bridges [40], [41]. Another study performed on live S2 cells, in which Top2 activity was reduced by either RNAi or chemical inhibition, did not detect defects in chromosome condensation and suggested that Top2 is required for centromere resolution and to prevent incorrect microtubule-kinetochore attachment [42]. Remarkably, downregulation of Top2 did not affect the mitotic index, indicating that Top2 deficiency does not activate cell cycle checkpoints in S2 cells [40], [42]. Other studies suggested that *Drosophila* Top2 is involved in homolog pairing in cell cultures [43], modulation of insulator function [44], and regulation of polytene chromosome structure [45].

Surprisingly, the role of Top2 in the maintenance of mitotic and meiotic chromosome structure in living flies has never been investigated. In a previous study we identified viable mutants in the *solofuso (suo)* gene. *suo^1^* and *suo^2^* mutant spermatocytes exhibit severely defective ana-telophases with extensive chromatin bridges; these telophases give rise to achromosomal secondary spermatocytes that are able to assemble bipolar spindles and divide in the complete absence of chromosomes [46]. Here we show that *suo^1^* and *suo^2^* are weak mutant alleles of the *Top2* gene and describe the isolation and characterization of a stronger *Top2* mutation. We show that modulation of Top2 function by means of these mutant alleles and in vivo RNAi results in multiple cytological phenotypes including site-specific chromosome aberrations, heterochromatin undercondensation, polyploidy, and complete disruption of chromosome morphology accompanied by a cell cycle arrest. These phenotypes recapitulate most of the phenotypes observed in vertebrate cells and indicate that *Drosophila* chromosomes are exquisitely sensitive to the residual level of Top2 in the cell.

## Results

### Isolation and characterization of *Top2* mutations

In a previous study we identified and characterized *suo^1^* and *suo^2^*, two viable but sterile mutants that exhibit many chromatin bridges and lagging chromosomes in male meiosis [46]. *suo* was originally mapped to the polytene interval 37C-37F5 uncovered by *Df(2L)VA17* [46]. Using the Exelis kit of deficiencies, we restricted this interval to the polytene band 37E1, uncovered by *Df(2L)Exel9043*, which contains four genes: *CG10237, RanGAP, Hs2st* and *Topoisomerase II (Top2)*. Complementation tests revealed that *suo* mutations are not allelic to either *CG10237* or *RanGAP*. However, sequencing of *Hs2st* and *Top2* in *suo* mutants did not reveal alterations in the protein coding exons of these genes with respect to those of another stock of the “Zuker collection” from which the *suo* mutants have been originally isolated (see Materials and Methods for detailed explanation). We thus screened a collection of 1,500 lethal mutations for those that fail to complement *suo^1^* (see Materials and Methods). We isolated a lethal mutation that in combination with *suo^1^* is semi-lethal and elicits the same meiotic phenotype observed in *suo* mutants (see below); this mutation was initially named *suo^3^*. DNA analysis of *suo^3^* mutants revealed a G-A transition at nucleotide 1,040 of the *Top2* coding sequence, corresponding to the 5′ splicing site of the second intron of the gene. This substitution results in a premature stop codon that would lead to a truncated form of the protein. These results indicate that *suo^3^* is allelic to *Top2*. Thus, we renamed *suo^1^, suo^2^* and *suo^3^* as *Top2^suo1^*, *Top2^suo2^* and *Top2^suo3^*, respectively. We note that we analyzed only the protein coding sequences of the *Top2^suo1^* and *Top2^suo2^* mutants but not the introns the UTRs or the 5′ regulatory sequences. Thus, the molecular lesions resulting in these mutations remain to be determined.

As previously reported, both *Top2^suo1^* and *Top2^suo2^* homozygous flies are viable but sterile in both sexes [46]. *Top2^suo3^* homozygous individuals die during embryonic development, but this early lethal phenotype is due to a second site mutation, as *Top2^suo3^/Df(2L)Exel9043* (henceforth *Top2^suo3^/Df*) animals survive until an early third instar larval stage. *Top2^suo3^/Df* larvae are devoid of imaginal discs like strong mitotic mutants [47] and exhibit small brains compared to controls at the same stage. *Top2^suo2^/Df* individuals are viable but male and female sterile; *Top2^suo1^/Df* and *Top2^suo1^*/*Top2^suo3^* mutant flies are semi-lethal, with a few escapers. Together these results allow the ordering of our *Top2* mutations in an allelic series with *Top2^suo3^*>*Top2^suo1^*>*Top2^suo2^*.

**Figure 1:**
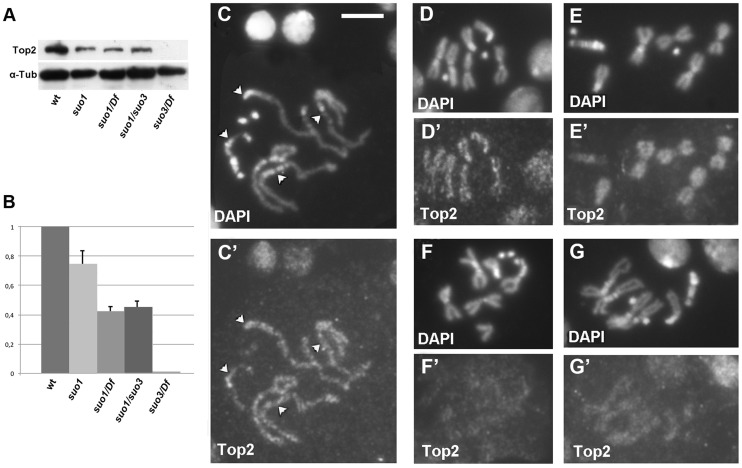
Top2 localization in wild type and *Top2* mutant larval brain cells. (A) Western blotting of brain extracts showing that the anti-Top2 antibody recognizes a 170 kDa band in control cells; this band is reduced in *Top2^suo1^/Top2^suo1^, Top2^suo1^/Df* and *Top2^suo1^/Top2^suo3^* mutants and undetectable in *Top2^suo3^/Df*. Tubulin was used as loading control. (B) Quantification of the relative intensities of the bands (± SD) from 4 different Western blotting experiments; the columns represent the relative ratios between the Top2 and the tubulin band intensities determined by densitometric analysis, with the wild type ratio set to 1. (C–E) Wild type male late prophase (C/C′), and female (D/D′) and male (E/E′) metaphases stained with the anti-Top2 antibody; note the discontinuous staining and the absence of Top2 accumulation at the kinetochores in D′ (arrowheads) and C′. In E′, the discontinuous staining is not visible but the centromeric regions of the major autosomes are clearly less stained than the other chromosomal regions. In *Top2^suo1^/Df* mutant brains (F/F′) and *Top2^suo1^/Top2^suo3^* (G/G′) chromosome immunostaining is reduced compared to wild type. Scale Bar, 5 µm.

To assess the amount of the Top2 protein in different mutant combinations, we performed a Western blot analysis on larval extracts using a polyclonal antibody directed against *Drosophila* Top2 [48]. In wild type extracts, this antibody recognized a single band of the expected size (170 kDa). The intensity of this band was reduced to approximately 80% of the control level in extracts from *Top2^suo1^* homozygous brains, showed further reduction in extracts from both *Top2^suo1^/Df* and *Top2^suo1^*/*Top2^suo3^* larvae, and was virtually undetectable in *Top2^suo3^/Df* extracts (Figure 1A, B). Immunostaining with an anti-Top2 antibody [49] showed that Top2 is enriched in interphase nuclei and mitotic chromosomes. In particularly favorable preparations we could observe a discontinuous Top2 distribution on chromosomes with alternating stained and unstained regions and no specific centromeric enrichments (Figure 1C–E). This staining pattern is consistent with that observed in vertebrate chromosomes in which the Topo II negative regions are more enriched in condensins compared with the Topo II positive regions [13], [14]. An alternating condensin/Top2 immunostaining pattern has been previously observed also in *Drosophila* S2 cells, although it was less sharply defined than in vertebrate chromosomes [42], [50]. In *Top2^suo1^/Df* and *Top2^suo1^/Top2^suo3^* brains immunostained for Top2, both mitotic chromosomes and interphase nuclei showed a weaker fluorescence compared with wild type (Figure 1F and G); the nuclei of *Top2^suo3^/Df* did not show any Top2 signal. These results agree with the genetic data and indicate that *Top2^suo1^* and *Top2^suo2^* are hypomorph alleles, while *Top2^suo3^* is a very strong and potentially null allele.

The availability of flies expressing different levels of Top2 allowed us to examine the roles of this protein in a physiological context. We focused on the phenotypes elicited by *Top2* mutants in three different *Drosophila* tissues: testes, brain and salivary glands.

### Top2 is required for proper chromatin organization within the nuclei of prophase I spermatocytes

During prophase I, *Drosophila* spermatocytes organize their chromatin in 3 main distinct clusters that localize at the periphery of the nucleus. Each of these clusters corresponds to one of the major *Drosophila* bivalents (X-Y, 2-2 and 3-3); the small fourth chromosome bivalent sometimes is separated from the three major chromatin clumps but more often associates with the X-Y bivalent. Before meiosis, chromosomes become progressively individualized within each chromatin territory but bivalents remain well separated from each other until metaphase I, when they congress at the center of the cell [51]–[54]. Previous work has shown that chromatin clumps morphology is affected by mutations in the condensin II coding genes and in genes that specify proteins required for homologous paring of the achiasmatic male chromosomes [55]–[58].

**Figure 2:**
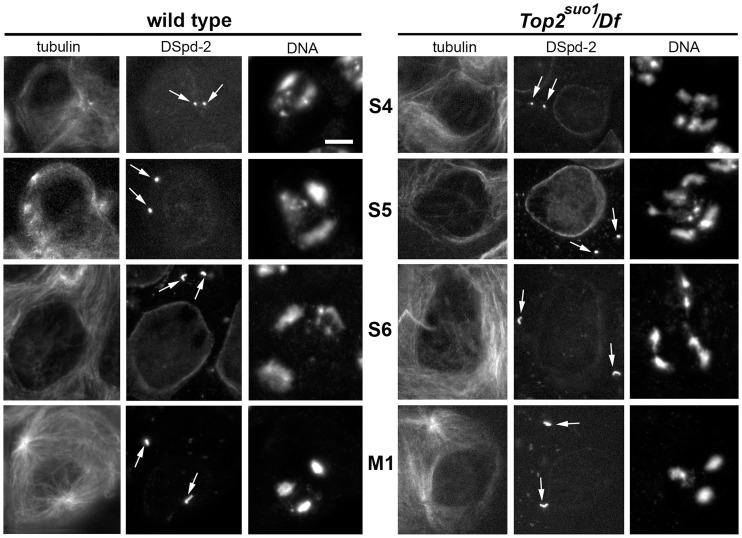
*Top2^suo1^/Df* spermatocytes exhibit an abnormal chromatin distribution within the prophase nuclei. Primary spermatocytes were stained for DNA, tubulin and the centriole marker DSpd-2 (arrows). Centriole size and morphology facilitated stage (see ref [52]) recognition and comparison between *Top2^suo1^/Df* and wild type cells. Scale Bar, 10 µm.

We thus analyzed chromatin organization within the prophase nuclei of *Top2^suo1^/Df* primary spermatocytes. To compare mutants and wild type spermatocytes, we stained cells for DNA (with DAPI), tubulin and the centriole marker DSpd-2 [59], and used centriole length as an additional criterion for stage identification (Figure 2). *Top2* spermatocytes at stages S2 and S3 were not very different from those of wild type (see ref [52] for stage description). However, at stage S4 they showed a higher number of distinct chromatin masses than wild type controls. This increase in chromatin masses was more evident in S5 spermatocytes; *Top2^suo1^/Df* spermatocyte nuclei displayed an average number of 5.6 chromatin masses per cell (n = 60) while control cells showed an average of 3.4 masses/nucleus (n = 60). Notably, in *Top2* mutant spermatocytes at stages S4 and S5 chromatin clumps of similar size were often closely apposed, suggesting that homologs were unpaired but remained in the vicinity of one another (Figure 2). In the subsequent stages of *Top2/Df* spermatocyte growth, the number of chromatin clumps progressively reduced. At prometaphase (stage M1), mutant spermatocytes displayed 3 compact clumps like wild type cells (Figure 2, and ref [46]); at metaphase (stage M3) they showed a central chromatin mass like wild type controls [46]. Collectively, these results indicate that during the early stages of spermatocyte growth Top2 is required for homolog co-mingling in a single nuclear territory. However, at later stages of spermatocyte development the homologs appear to pair through a Top2-independent mechanism.

### *Top2* mutant males exhibit severe defects in meiotic chromosome structure and segregation

**Figure 3:**
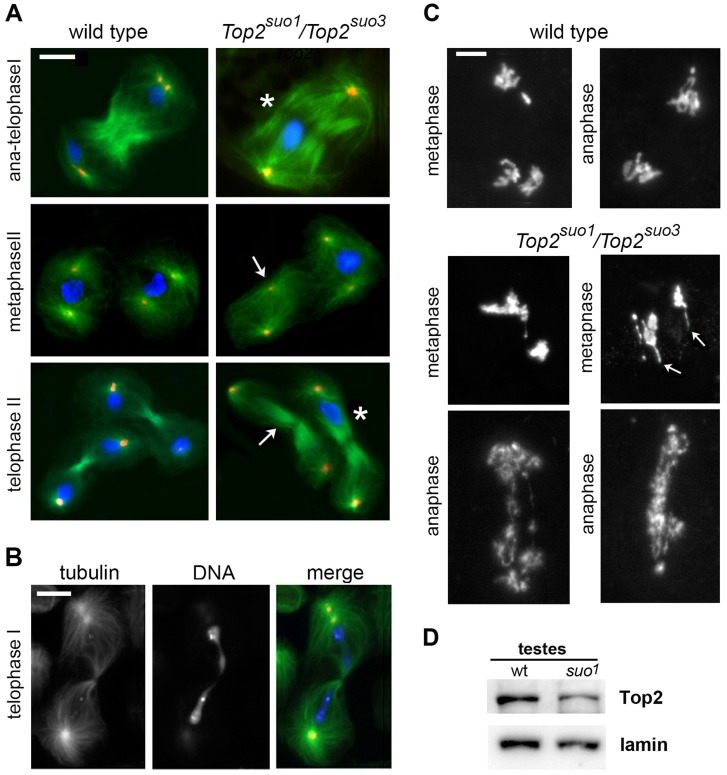
*Top2^suo1^/Top2^suo3^* mutant males display strong defects in meiotic chromosome morphology and segregation. (A, B) Cells were fixed with methanol/acetone (see materials and methods) and stained for tubulin (green), Dspd-2 (red), and DNA (DAPI, blue). In the mutant telophases I and II marked with an asterisk (A), all chromosomes are segregating to one of the daughter cells; in another telophase I (B), a long chromatin bridge connects the two sets of segregating chromosomes. As a consequence of the aberrant chromosome segregation during the first meiotic division, many spindles of the second division are chromosome-free (arrows). Scale Bars, 10 µm. (C) Wild type and *Top2^suo1^/Top2^suo3^* meiosis I figures fixed with 45% acetic acid and stained with DAPI. The *Top2^suo1^/Top2^suo3^* metaphase chromosomes appear as chromatin masses in which the individual chromatids are no longer recognizable. Note the thin chromatin protrusions (arrows) in mutant metaphases, and the aberrant chromosome structure and segregation in mutant anaphases. Scale Bars, 5 µm. (D) Western blot showing that testes from *Top2^suo1^/Top2^suo1^* males exhibit a reduction in the Top2 level with respect to wild type. Densitometric analysis of the bands from 3 different blots revealed that this reduction is of 50±5%. Lamin was used as loading control.

Previous studies characterized the meiotic phenotype of mutant males homozygous or hemizygous for *Top2^suo1^* or *Top2^suo2^* [46]. Here we examined the meiotic phenotype of flies bearing the *Top2^suo3^* allele after staining for tubulin, DSpd-2 and DNA. In *Top2^suo3^*/*Df* testes, no meiotic divisions were found due a severe defect in germ cell proliferation. The meiotic phenotype of *Top2^suo1^/Top2^suo3^* males was similar to that of *Top2^suo1^/Df* males, with all ana-telophase I figures characterized by highly defective chromosome segregation and chromatin bridges (Figure 3A, B). As previously described, in approximately one half of these aberrant ana-telophases, all chromosomes segregated to a single pole, giving rise to secondary spermatocytes that divided in the complete absence of chromosomes. Secondary spermatocytes that received some chromosomes also displayed extensive defects in chromosome segregation with frequent chromatin bridges (Figure 3A; see also ref [46]). These results demonstrate that Top2 is required for chromosome segregation during both meiotic divisions of *Drosophila* males, highlighting a crucial role of the protein not only in sister chromatid separation but also in bivalent segregation.

Because the methanol-based fixation technique used to preserve spindle structure results in poorly defined chromosomes, we fixed larval testes in 45% acetic acid to maintain proper chromosome morphology (see Materials and Methods). The chromosomes of all metaphase and early anaphase I figures of *Top2^suo1^/Df* and *Top2^suo1^/Top2^suo3^* spermatocytes displayed very severe structural defects. In metaphase figures, bivalents appeared as chromatin masses in which individual sister chromatids were no longer discernible (Figure 3C). Interestingly, approximately 60% of these aberrant metaphase figures (n = 18) showed one or more long chromatin protrusions that were not observed in wild type spermatocytes (Figure 3C). Similar protrusions have been observed in Top2-depleted meiotic cells of *Drosophila* females; these protrusions emanated from the chromatin mass of metaphase I figures and contained the centromeres at their tips, suggesting that they were generated by the pulling forces exerted by the spindle (Hughes and Hawley; cosubmitted). In anaphase-like figures, individual chromosomes were also no longer recognizable, and the two presumptive daughter cells were almost invariably connected by a number of entangled and irregularly condensed chromatin threads (Figure 3C). These entangled threads resulted in long ana-telophase chromatin bridges suggesting that the kinetochores of the entangled chromosomes are pulled away by the spindle microtubules (Figure 3B and 3C). The different appearance of metaphase and anaphase chromosomes is a likely consequence of the spindle pulling forces, which would stretch and partially disentangle the metaphase chromosomes. However, in approximately 50% of the cases the chromosome tangles generated by loss of *Top2* function were not resolved and gave rise to telophase figures in which all chromosomes remained in one of the daughter cells (Figure 3A).

### *Top2* is required for mitotic chromosome integrity

**Figure 4:**
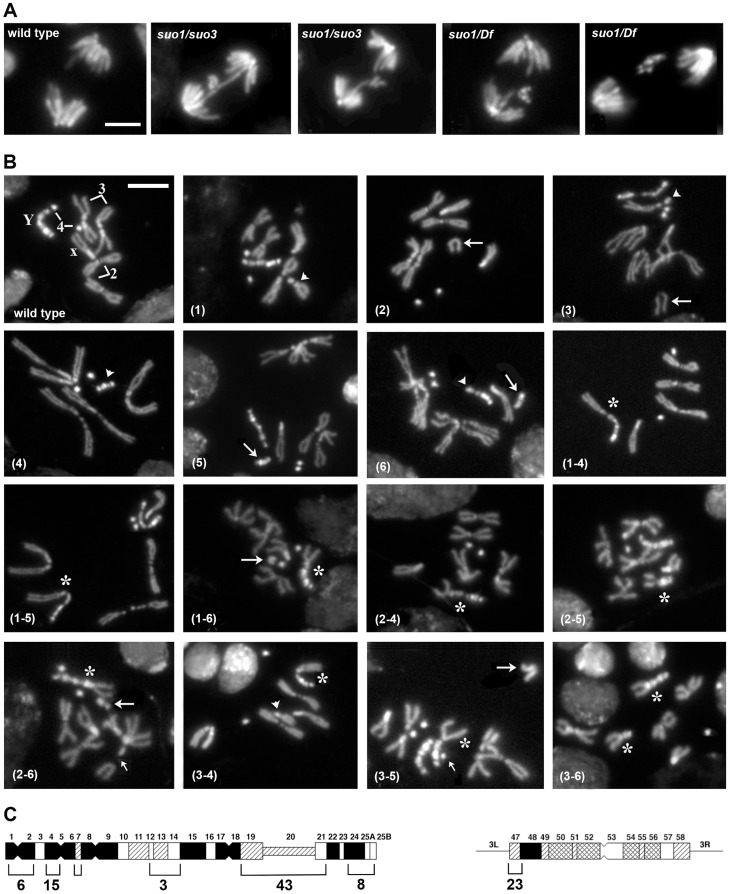
The site-specific CABs observed in *Top2^suo1^/Top2^suo3^* mutant brains are generated by bridge severing during anaphase. (A) Examples of wild type and *Top2* mutant anaphase figures with bridges and lagging acentric fragments. Scale Bar, 5 µm. (B) Examples of aberrations generated by chromosome breaks that occurred during the anaphase of the previous cells cycle. Panels 1–3 show metaphases carrying a broken third chromosome without the corresponding acentric fragment (1), a complete chromosome complement with an extra acentric fragment (2), and a complete isochromatid deletion with a breakpoint in region h47 of 3L heterochromatin; note that at one of the ends of the acentric fragments shown in panels 2 and 3 the sister chromatids are closely apposed as occurs in the heterochromatic regions. The aberrant metaphases shown in panels 1–3 are likely to be generated by anaphase events leading to the G1 configurations depicted in Figure 5. Panels 4–6 show Y chromosome aberrations resulting from duplication of the broken G1 Ys depicted in Figure 5. The remaining panels show chromosome exchanges between the Y chromosome and the 3L heterochromatin generated by interactions between broken G1 chromosomes, as illustrated in Figure 5. For example the Y-3 dicentric chromosome shown in panel (1–4) is generated by fusion of the broken ends of the G1 chromosomes in the 1 and 4 “cells” of Figure 4. In all panels, arrowheads and arrows point to centric and acentric chromosome fragments, respectively; the asterisks indicate dicentric and translocation chromosomes involving the Y and 3L heterochromatin. The small arrows in panels (2–6) and (3–5) point to an additional break in 3L heterochromatin and an extra Y fragment, respectively. Scale Bar, 5 µm. (C) Distribution of 77 Y chromosome breakpoints and 23 third chromosome breakpoints of selected dicentrics and translocations. The heterochromatin maps are from Gatti and Pimpinelli [60]. The numbers above and below the maps indicate the different heterochromatin regions and the numbers of breakpoints found in these regions, respectively.

To address the controversial role of Topo II in the maintenance of proper mitotic chromosome architecture, we first analyzed *Top2^suo1^/Top2^suo1^, Top2^suo1^/Df* and *Top2^suo1^/Top2^suo3^* brains fixed without hypotonic and colchicine pre-treatments. In contrast with the spermatocyte chromosomes, the chromosomes of *Top2^suo1^/Top2^suo1^* mutant brains were morphologically normal. Chromosome morphology was substantially normal also in *Top2^suo1^/Df* and *Top2^suo1^/Top2^suo3^* mutant brains; we observed only a few cells (∼5%) showing a mild undercondensation of the heterochromatic regions of the major autosomes but the appearance of euchromatic arms was always normal (Figure 4). To determine whether the chromosomal phenotypes observed in spermatocytes and brain cells were due to different levels of Top2 we prepared testis extracts from *Top2^suo1^/Top2^suo1^* mutants. Western blotting analysis showed these extracts exhibit a ∼50 reduction in the Top2 abundance. This reduction is comparable to that observed for *Top2^suo1^/Df* brains (∼44%; Figure 1A) in which the chromosome morphology is essentially normal. This suggests that spermatocyte and brain chromosomes require different levels of Top2 activity to achieve a regular structure, and that spermatocyte chromosomes are particularly sensitive to Top2 depletion.

In *Top2^suo1^/Top2^suo1^* mutant brains fixed without colchicine and hypotonic pre-treatments, the mitotic index (MI, the proportion of cells engaged in mitosis; see Materials and Methods) and the frequency of anaphases were comparable to those of wild type brains (Table 1). *Top2^suo1^/Df* brains also showed a normal MI but displayed a significant increase in the frequency of anaphases with respect to both control and *Top2^suo1^/Top2^suo1^* brains. This finding is likely to reflect a delay in progression through anaphase of mutant cells. In addition, 37% of the anaphases observed in *Top2^suo1^/Df* brains displayed chromatin bridges and/or acentric fragments that failed to correctly segregate (Figure 4 A). However, we never observed entire lagging chromosomes with unseparated sister chromatids. Thus, in *Top2^suo1^/Df* brains centromere resolution does not appear to be affected as occurs in Top2-depleted S2 cells [42].

**Table 1:** Mitotic parameters in *Top2* mutants.

Genotype	# of brains	# of fields	# of mitotic figures	Mitotic index (MI)	Anaphases (%)	Irregular anaphases (%)
wt	12	643	527	0.82	17.1	0
*suo^1^/suo^1^*	10	576	456	0.79	18.9	3.5
*suo^1^/Df*	11	596	550	0.92	25.1	37.0

*Top2* mutant brains fixed without colchicine and hypotonic pre-treatments also displayed several chromosome aberrations (CABs). To define the type and frequency of CABs elicited by the different *Top2* mutant alleles we incubated dissected brains in saline with 10^−5^ M colchicine for 1 hour and treated them with hypotonic solution before fixation. Colchicine arrests mitotic cells in metaphase and hypotonic treatment results in chromosome spreading facilitating CAB scoring. In *Top2^suo2^/Top2^suo2^* brains the CAB frequency was not significantly higher than that of wild type controls, which exhibit 0.008 CABs per cell (Table 2). *Top2^suo1^/Top2^suo1^* mutant brains showed approximately 0.04 CABs/cell, while brains of both *Top2^suo1^/Df* and *Top2^suo1^/Top2^suo3^* larvae displayed CAB frequencies ranging from 0.25 to 0.53 per cell (Figure 4B; Table 2). An analysis of the CABs observed in *Top2^suo1^/Df* and *Top2^suo1^/Top2^suo3^* brain metaphases reveled a striking specificity. In mutant males, most CABs were isochromatid breaks that preferentially involved the entirely heterochromatic Y chromosome (48.3% of isochromatid breaks) and the third chromosome heterochromatin (46.1%). In addition, mutant males displayed 18–19% chromosome exchanges, most of which (∼98%) were dicentrics or translocations generated by breaks in the Y chromosome and 3L heterochromatin (Figure 4B). A similar CAB pattern was observed in XXY females in which 76% of the aberrations involved the Y chromosome, the third chromosome heterochromatin or both (Table 2). Examination of selected rearrangements displaying particularly clear heterochromatin banding revealed that the Y and third chromosome breakpoints are non-randomly distributed; the third chromosome breakpoints were clustered in region h47 of 3L heterochromatin, while the Y breakpoints were localized in several regions with a preference for regions h4-h5 and h19-h22 (Figure 4B and C; see ref [60] for a cytological map of *Drosophila* heterochromatin). In *Top2^suo1^/Df* and *Top2^suo1^/Top2^suo3^* XX females most of the CABs were isochromatid breaks in region h47 of 3L heterochromatin. In contrast with males, these females did not display chromosome exchanges (Table 2). However, should these exchanges occur between the third chromosomes and involve the heterochromatic regions, they would be hard to detect as they would appear as intact chromosomes 3.

**Table 2:** Types and frequencies of chromosome aberrations (CABs) observed in *Top2* mutant brains.

Genotype	# of cells scored	Cd	Complete iso %			Iso no F (%)			Extra F (%)			Exchanges (%)			Total CABs (%)	TFs (%)
			Y	3Lh	Oth	Y	3Lh	Oth	Y	3Lh	Oth	Y-3Lh	Y-oth	Non Y		
*ywf* f	800	0.5	-	0	0.4	-	0	0	-	0	0	-	-	0	0.9	
*ywf* m	600	0.3	0	0	0.5	0	0	0	0	0	0	0	0	0	0.8	0
*suo^2^/suo^2^* f	200	0.5	-	0	0.5	-	0	0	-	0	0	-	-	0	1.0	0
*suo^2^/suo^2^* m	240	0.4	0	0	0.4	0	0	0	0	0	0	0	0	0	0.8	0
*suo^1^/suo^1^* f	210	0	-	0.9	0.5	-	0.9	0	-	0.5	0.5	-	-	0	3.3	0
*suo^1^/suo^1^* m	594	0.3	0.7	0.7	0.3	0.3	0.7	0	0.2	0.3	0.3	0.3	0	0	4.1	0
*suo^1^/Df* f	528	1.9	-	7.4	3.6	-	5.7	1.5	-	2.8	1.5	-	-	0.6	25.0	0
*suo^1^/Df* m	349	0.9	4.9	3.4	2.6	2.9	3.1	1.1	2.0	2.0	1.7	16.9	1.7	0.3	43.5	0
*suo^1^/suo^3^* f	245	0.8	-	7.8	2.0	-	6.1	1.2	-	3.7	2.9	-	-	0.8	25.3	0
*suo^1^/suo^3^* m	539	0.7	3.5	4.3	3.3	3.3	3.1	2.0	2.8	2.2	1.9	15.8	1.9	0.2	45.0	0
*suo^1^/suo^3^ XXY*	210	1.4	4.8	2.9	4.3	5.2	3.3	2.9	2.9	2.4	1.9	19.0	2.4	0	53.4	0
*mei41* m	300	2.0	0	0	3.0	0	0	0.3	0	0	0	0	0	0	5.3	0
*mei41; suo^1^/suo^3^* m	318	2.5	7.9	3.5	2.2	7.2	5.3	1.3	5.7	4.4	3.5	27.4	6.9	0.6	78.4	0
*tefu/tefu* m	249	4.8	0	0	4.0	0	0	0.4	0	0	0.4	0.4	0	0	10.0	56.6
*tefu/tefu; suo^1^/suo^3^* m	474	4.0	4.9	4.2	3.2	4.4	3.4	1.3	2.9	1.9	2.1	12.2	2.1	1.9	48.5	50.6

m, males; f, females; Cd, chromatid deletions; iso, isochromatid deletions; F, chromosome fragment; TFs, telomere fusions; 3Lh, 3L heterochromatin; Oth, other (chromosome regions different from Y or 3L heterochromatin).

Importantly, in 65% of the mutant metaphases with a broken Y chromosome the acentric fragment was either missing or present as an additional element to a normal chromosome complement (the Y is easily recognized for its brightly fluorescent bands; see Figure 4). Similarly, 35% of the cells with a broken third chromosome showed only the centric portion of the chromosome. We also observed many cells (20%) with a normal chromosome complement and an extra acentric fragment of the size and the appearance of an entire 3L euchromatic arm. We thus assume that this acentric fragment is the exact complement of the centric element broken within 3L heterochromatin. Cells carrying either an isocromatid break lacking the acentric fragment or a normal chromosome complement plus an extra acentric fragment are likely to be generated during the anaphase of the previous cell cycle by the breakage of bridges between entangled chromatids.

**Figure 5:**
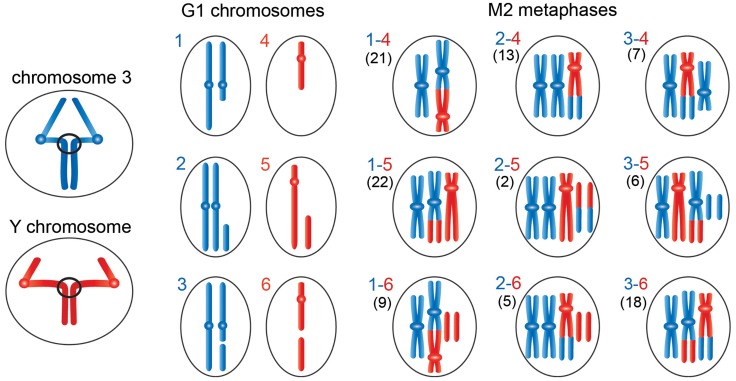
Schematic representation of the events leading to the chromosome rearrangements shown in Figure 3. The panels on the left show third (blue) and Y (red) chromosomes undergoing anaphase with entangled sister chromatids. The G1 panels show the results of ruptures of the chromatin bridges generated by the anaphase entanglements; we considered breaks involving only one of the two chromosomes 3. During anaphase the acentric fragment could be lost (outcome in schemes 1 and 4), segregate at the cell pole that contains an intact chromosome complement (outcome in schemes 2 and 5), or segregate with the centric element of the broken chromosome (outcome in schemes 3 and 6). The other panels show the types of Y-3 exchanges that could be generated by the chromosome breaks depicted in panels 1–6. The exchanges generated by 1–4, 1–5, 2–4 or 2–5 interactions have unique configurations (see Figure 4); 1–6, 2–6, 3–4, 3–5 or 3–6 interactions can give rise to different type of exchanges. For example, 3–4 interactions can produce the half translocation depicted here and shown in Figure 4 or the dicentric chromosome shown Figure S2. Similarly, 3–6 interactions can produce the translocation shown here and in Figure 3 or the dicentric chromosome shown in figure S2. We classified 103 Y-3 exchanges; the numbers of the different types of rearrangements observed are reported between parentheses on the left of each diagram.

The bridges observed in *Top2* mutant anaphases are likely to involve sister rather than nonsister chromatids because the analysis of 100 anaphase figures revealed that the bridges, or the acentric fragments generated by their rupture, never involve a banded Y chromatid and a uniformly stained autosomal or XL chromatid (Figure 4A and S1). Thus, although we cannot formally exclude that some of the evenly stained bridges/fragments are generated by entanglements between nonsister chromatids, we assume most bridges observed in *Top2* mutant anaphases are due to aberrant associations between the sister chromatids of either the Y or the third chromosome (Figures 4 and 5). As shown in Figure 4B (panels 1–6) and depicted in Figure 5, the acentric fragment resulting from the rupture of a bridge is subject to three different fates: it could be lost, co-segregate with its intact sister chromatid, or co-segregate with its complementary centric fragment.

The hypothesis that incomplete isochromatid breaks are generated during the anaphase of the previous cell cycle is supported by the analysis of 103 chromosome exchanges (selected on the basis of their cytological quality) involving the Y and 3L heterochromatin. Indeed, as shown in Figures 4 and 5, the majority (82%) of these rearrangements involve at least one element that can only be generated by the rupture of an anaphase bridge: either a centric fragment devoid of its acentric companion (panels 1 and 4 of Figures 4B and 5) or an acentric fragment accompanied by its intact homologue (panels 2 and 5 of Figures 4B and 5). Interactions between these elements and between them and the two complementary fragments of a broken chromosome (which are also likely to be generated during anaphase; see panels 3 and 6 of Figures 4B and 5) would result in the 9 types of chromosome rearrangements shown in figure 4B and depicted in Figure 5. In Figure 5, we depicted only the outcomes of single breaks in the Y and/or in one of the two third chromosomes. We did not consider the rearrangements generated by breaks in both chromosomes 3 or by two breaks in the Y chromosome. However, these complex CABs are rather rare and represent only 3% of the rearrangements involving the Y and/or the third chromosome; some examples of these complex rearrangements are shown in figure S2.

An analysis of the frequencies of the various types of rearrangements permits us to pinpoint the anaphase events that led to their formation. As shown in Figure 5, the rearrangements that include centric Y and third chromosome elements with no complementary acentric fragments are 41 and 52, respectively; those that contain intact Ys or third chromosomes plus extra fragments are 30 and 20, respectively; and those including centric Y and third chromosome elements plus the respective complementary fragments are 32 and 31, respectively. Given that the cells contain two chromosomes 3 and a single Y, this analysis establishes that the Y is more frequently involved in anaphase breaks than the third chromosome, consistent with the fact that the Y is breakable in multiple sites and the third chromosome in a single site. In addition, it appears that the most frequent anaphase event that will subsequently generate chromosome exchanges is the transmission to one of the two daughter cells of a centric element not accompanied by its complementary fragment. These findings are consistent with the observed frequencies of isochromatid breaks (Table 2). However, the relative frequencies of complete isochromatid breaks (centric element plus complementary fragment; Table 2) are slightly higher than those derived from the analysis of the exchanges. The simplest explanation for this discrepancy is that the anaphase events leading to the chromosomal configurations depicted in Figure 4 are not completely independent, so that the frequencies of double events is not the one expected based on independency of single events. In summary, our findings strongly suggest that most CABs observed in *Top2^suo1^/Df* and *Top2^suo1^/Top2^suo3^* mutants derive from site-specific breaks generated during the anaphase of the previous cell cycle.

### Top2 is required for cell cycle progression

**Figure 6:**
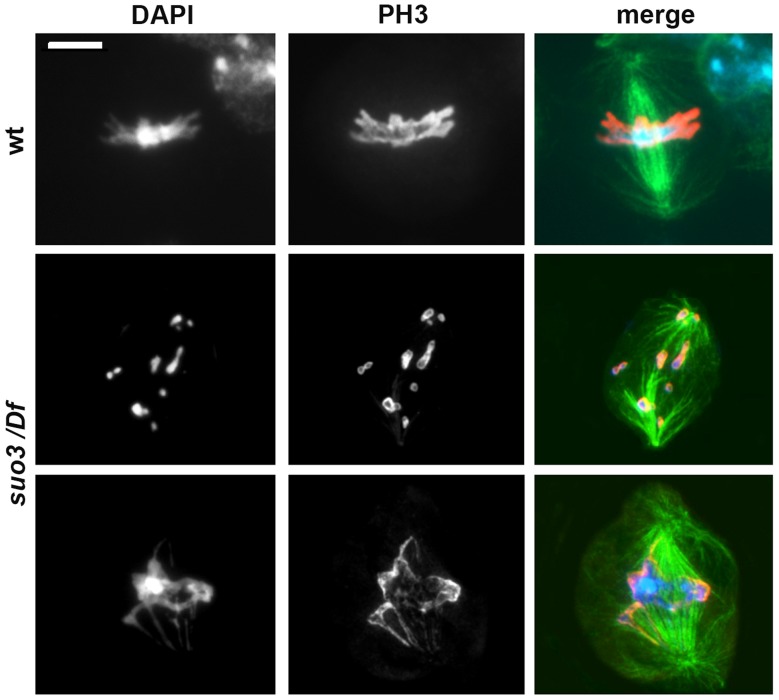
Examples of the rare mitotic metaphases observed in *Top^suo3^/Df* mutants. Cells were stained for DNA (DAPI, blue in merges), mitotic phosphohistone (red in merges) and tubulin (green in merges); wt, wild type. Scale Bar, 5 µm.

While *Top2^suo1^/Df* and *Top2^suo1^/Top2^suo3^* mutant cells appear to progress almost normally through mitosis despite the presence of CABs, *Top2^suo3^*/*Df* brains displayed a dramatic drop in the MI. In brains stained only with DAPI we were unable to observe clear mitotic figures. We thus analyzed *Top2^suo3^*/*Df* brains stained with DAPI and immunostained for both tubulin and the mitotic H3 phospho-histone, which marks mitotic chromatin [61]. In 25 brains examined, we observed only 10 metaphase-like figures (a single wild type brain usually contains 50–100 mitotic figures). The chromosomes of these rare metaphases appeared as chromatin masses in which individual chromosomes and sister chromatids were no longer recognizable (Figure 6). However, despite these severe chromosomal defects, all metaphases showed bipolar spindles with relatively normal microtubule densities. Growing evidence indicates that *Drosophila* somatic cells require kinetochore-driven MT growth for correct bipolar spindle formation [62], [63]. Thus, the observation that *Top2^suo3^*/*Df* cells can assemble a normal bipolar spindle suggests that Top2-depleted chromosomes/kinetochores retain the ability to drive MT nucleation.

The extremely low MI observed in *Top2^suo3^*/*Df* brain preparations might be the consequence of a checkpoint that prevents cells to progress through the cell cycle and enter mitosis. To ask whether the interphase arrest of Top2-deficient cells is caused by checkpoint activation we examined *mei-41; Top2^suo3^*/*Df* and *Top2^suo3^*/*Df*; *tefu/tefu* double mutants. *mei-41* and *tefu* are the *Drosophila* orthologues of *ATR* and *ATM*, respectively; the kinases encoded by these genes are involved in the signaling pathway of the S and G2-M DNA damage checkpoints [64], [65], and ATR is thought to mediate the DNA decatenation checkpoint [35]. We found that neither double mutant displays a MI higher than that seen in *Top2^suo3^*/*Df* mutants (in all cases we examined more than 20 brains and observed less than one mitotic cell per brain). Thus, neither ATR nor ATM loss was able to rescue the block in mitotic progression in *Top2^suo3^*/*Df* brains.

To obtain further insight into the type of DNA lesions caused by Top2 deficiency we examined brains from *mei-41; Top2^suo1^/Top2^suo3^* and *Top2^suo1^/Top2^suo3^*; *tefu/tefu* larvae. *mei-41; Top2^suo1^/Top2^suo3^* brains showed a substantial increase in the frequency of CABs compared to either single mutant (Table 2). In doubly mutant males, the relative frequencies (with respect to the total number of CABs) of isochromatid deletions involving the Y chromosome or the third chromosome heterochromatin and the relative frequencies of Y-3 exchanges were comparable to those found in *Top2^suo1^/Top2^suo3^* mutants. This suggests that downregulation of *mei-41* (ATR) function might either increase the frequency of anaphase entanglements generated by Top2 deficiency or inhibit repair of the site-specific chromosome breaks they produce. Previous studies have shown that mutations in *tefu* (ATM) cause both telomeric fusions (TFs) and CABs [66]. *Top2^suo1^/Top2^suo3^*; *tefu/tefu* brains displayed frequencies of TFs and CABs that appear to be the sum of those observed in *Top2^suo1^/Top2^suo3^* and *tefu/tefu* mutants. Thus, we conclude that *tefu* (ATM) and *Top2* function in different pathways that mediate maintenance of chromosome integrity.

### Analysis of the mitotic phenotype induced by in vivo *Top2* RNA interference

**Figure 7:**
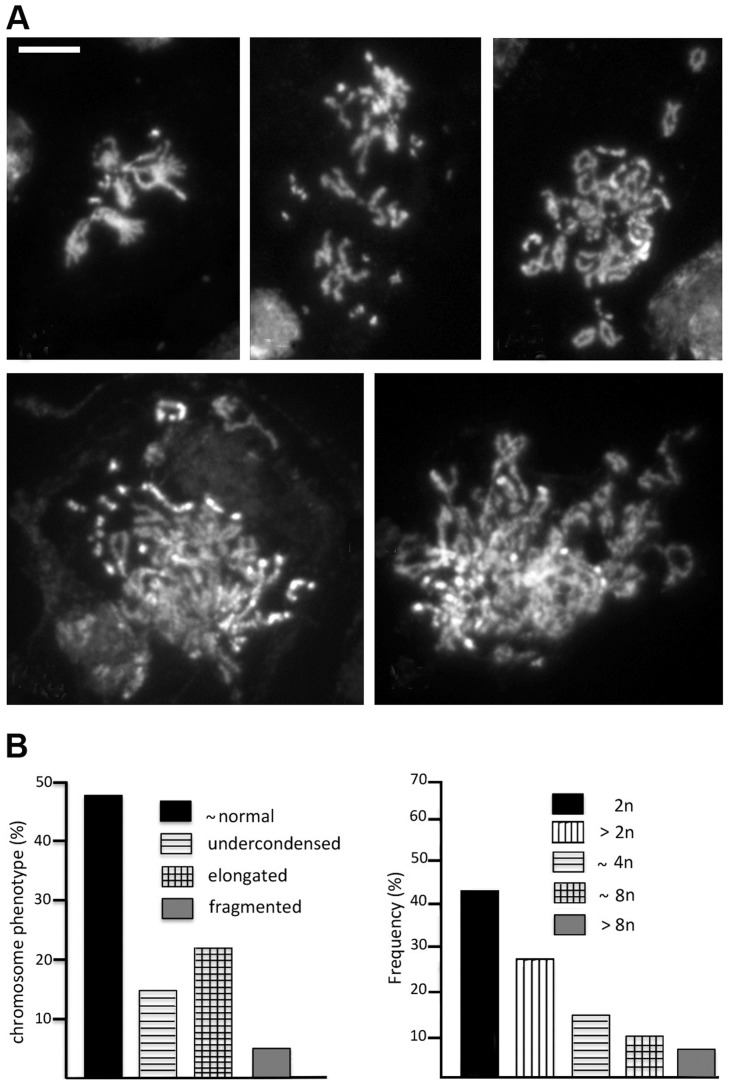
RNAi against *Top2* disrupts mitotic division in larval brains. (A) Examples of hyperploid and polyploid cells found in *Top2* RNAi brains. The panel in the upper left shows a hyperploid metaphase with tightly paired homologs; the other panels show polyploid metaphases with many broken chromosomes. Note that several breaks appear to involve the centric heterochromatin of the major autosomes; they could be either real interruptions in chromosome continuity or drastic failures in heterochromatin condensation. Scale Bar, 5 µm. (B) Frequencies of the various types of aberrant mitotic cells observed in *Top2* RNAi brains (n = 164).

The *Top2* mutants analyzed here showed different mitotic phenotypes. *Top2^suo1^/Df* and *Top2^suo1^/Top2^suo3^* brain cells showed a normal MI and frequent CABs that preferentially involve the Y and the 3L heterochromatin. In contrast, *Top2^suo3^/Df* brains showed a drastically reduced MI and rare metaphases with collapsed and/or shattered chromosomes. *Top2^suo1^/Df* and *Top2^suo1^/Top2^suo3^* brains contain ∼60% less Top2 than wild type brains, and Top2 is undetectable in *Top2^suo3^/Df* brains (Figure 1A). This suggests that larval brains with intermediate Top2 levels would produce intermediate phenotypes that would provide additional information on Top2 function. Thus, we analyzed the phenotype produced by RNAi in flies bearing an inducible *UAS-Top2* RNAi construct. Animals bearing this construct and an Actin-GAL4 driver died as third instar larvae; the brains of these larvae displayed a Top2 content that was barely detectable in Western blots but definitely higher than that of *Top2^suo3^/Df* larvae (Figure S3). Consistent with this finding, the mitotic phenotype of *Top2* RNAi brains was milder than that observed in *Top2^suo3^/Df* brains. The frequency of mitotic figures in brains stained for DNA, mitotic phosphohistone and tubulin was still very low (an average of 7 mitotic figures per brain; 23 brains examined) but basic chromosome morphology was maintained. The analysis of chromosome preparations from colchicine-treated and acetic acid-fixed brains revealed a phenotype that was not previously observed in *Top2^suo1^/Df* or *Top2^suo3^/Df* mutants. Most metaphases were either polyploid (31%) or hyperploid (27%) and showed extensive chromosome breakage (Figure 7A and B). These metaphases showed many free autosomal arms generated either by breakage or drastic undercondensation of centric heterochromatin (Figure 7A). We estimated that the frequency of these free arms was approximately three-fold higher than the frequency of breaks in euchromatin. This finding, together with the fact that the Y chromosome was invariably broken in multiple fragments or rearranged, suggests that in *Top2* RNAi cells most CABs involve heterochromatin.

The presence of hyperploid and polyploid metaphases in Top2-depleted brains suggests that these aberrant cells experienced nondisjunction events or complete cell division failures in the previous cell cycles. We also observed several prometaphase figures in which the homologous chromosomes were as tightly paired as in control cells (Figure 7A), suggesting that Top2 activity is not essential for somatic pairing in larval brain mitotic cells.

### Analysis of polytene chromosomes in *Top2* mutants

**Figure 8:**
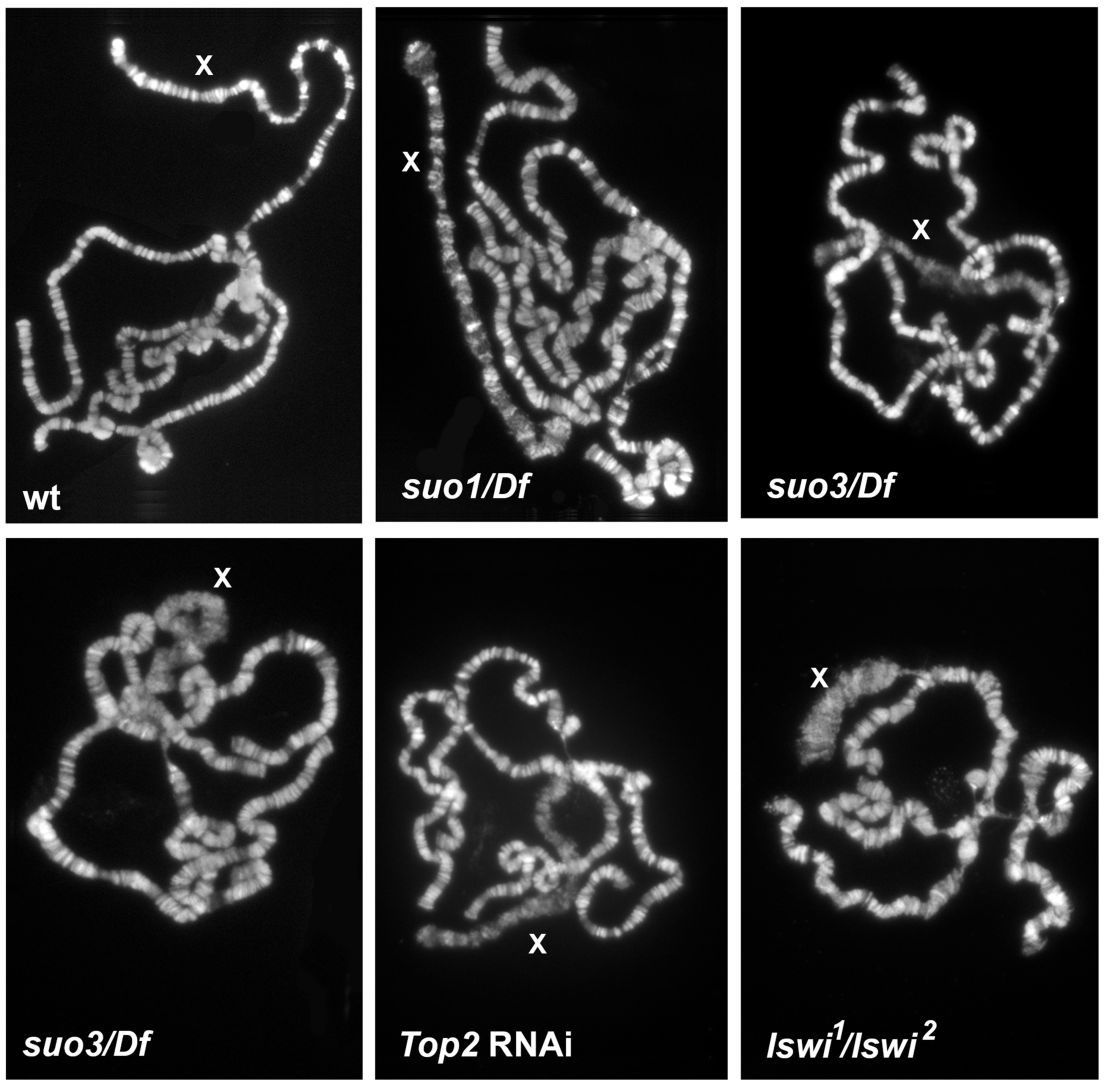
Mutations in *Top2* affect the X chromosome structure in male polytene nuclei. The polytene chromosomes of *Top2^suo1^/Df* males exhibit an X chromosome that maintains its banded appearance but is slightly bloated compared to wild type (wt). The polytene chromosomes of *Top2^suo3^/Df* and *Top2* RNAi males display very similar defects; they show a specific bloating and shortening of the X chromosome accompanied by an almost total loss of its banded structure. Note that the X chromosome morphology in *Iswi* mutants is very different from that seen in *Top2* mutants.

Examination of salivary glands revealed a role of Top2 in the control of polytene chromosome structure. In polytene preparations from *Top2^suo1^/Df, Top2^suo1^/Top2^suo3^* and *Top2^suo3^/Df* males we consistently observed a specific alteration in the morphology of the X chromosome, which appeared bloated and often detached from the chromocenter (Figure 8 and Figure S4). In *Top2^suo1^/Df* and *Top2^suo1^/Top2^suo3^* males, this deformed X chromosome conserved a banded appearance but its bands were less sharp than those of either the autosomes of the same nucleus or an X chromosome from wild type males. In general, the X chromosome condensation phenotype observed in *Top2^suo1^/Df* and *Top2^suo1^/Top2^suo3^* mutants was rather variable but clearly visible in all polytene nuclei. A more severe defect was consistently observed in *Top2^suo3^/Df* and *Top2* RNAi males, which showed a specific bloating and shortening of the X chromosome accompanied by a partial or total loss of its typical banding pattern (Figure 8 and Figure S4). In contrast, *Top2^suo1^/Df, Top2^suo1^/Top2^suo3^* and *Top2^suo3^/Df* females displayed morphologically normal polytene chromosomes. These results indicate that Top2 is required for proper chromatin organization of the dosage-compensated X chromosome of males. We note that the defect in X chromosome organization and condensation observed in *Top2^suo3^/Df* and *Top2* RNAi males is quite different from that caused by Cap-H2 (condensin) overexpression; an excess of Cap-H2 caused a tremendous axial compaction of all arms of polytene chromosomes [67], which was never observed in the X chromosome of *Top2* mutants.

**Figure 9:**
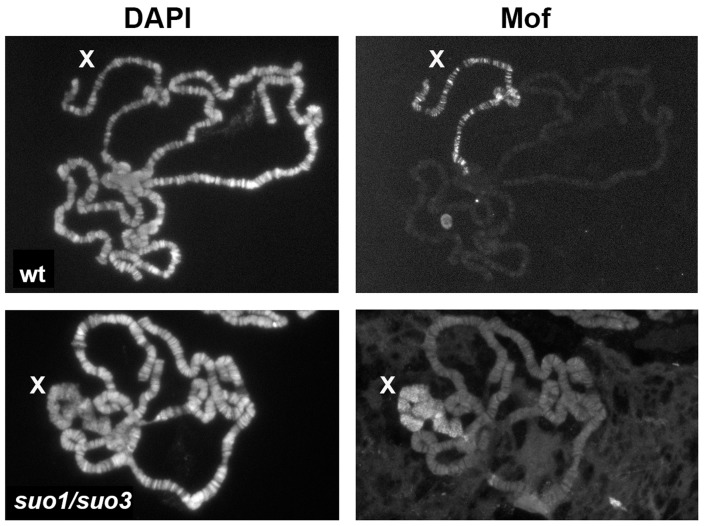
The X chromosome of polytene nuclei from *Top2* mutant males binds Mof. Note that Mof specifically decorates the X chromosome of both wild type and *Top2^suo1^/Top2^suo3^* males; wt, wild type.

Defects in the X chromosome condensation were previously observed in male polytene nuclei of *Imitation switch (ISWI)*, *Su(var)2-5* (HP1) and *Su(var)3-7* mutants [68]–[70]. HP1 and Su(var)3-7 are interacting proteins with multiple roles in *Drosophila* chromatin regulation [71], [72]; ISWI is the ATPase subunit of several chromatin remodeling complexes including CHRAC, ACF and NURF [73]. Dosage compensation and the architecture of compensated chromatin depend on the Male Specific Lethal (MSL) complex, which includes Msl1, Msl2, Msl3, Mle, the Mof acetyltransferase and the roX1 and roX2 noncoding RNAs (see refs [74] and [75] for review). Previous studies have shown that blocking H4k16 acetylation completely rescues the X chromosome condensation defects caused by *ISWI, Su(var)2-5* and *Su(var)3-7* mutations [70], [76], suggesting that histone acetylation modulates chromatin compaction of the male X chromosome. Based on these results, we investigated the relationships between Top2 and the dosage compensation system. Previous work has shown that the X chromosome of *Top2* mutants binds the Msl1 and Mle proteins [45]. Our immunostaining experiments showed that the bloated X chromosome of *Top2* mutants also binds the dosage compensation factor Mof (Figure 9). In addition, we found that the bloated appearance of the X chromosome of *Top*2 mutants is completely rescued in *mof*; *Top2* double mutants (Figure S5).

Although mutations in *ISWI* and *Top2* both affect X chromosome structure in male polytene nuclei, they do not result in identical phenotypes. A direct comparison between the polytene chromosomes of the mutants revealed that the X chromosome of *ISWI* mutants is shorter and more compact than those of either wild type or *Top2* mutant larvae (Figure 8). Because previous studies have suggested that Top2 and ISWI are both contained in the CHRAC remodeling complex [77], we asked whether the male X chromosome deformation caused by *Top2* mutations is a consequence of a failure to recruit ISWI at polytene chromosomes. Immunostaining of salivary glands from *Top2^suo1^/Top2^suo3^* males showed that an anti-ISWI antibody decorates the X chromosome, although with a diffuse pattern reflecting the altered architecture of the chromosome (Figure 9). However, immunostaining of salivary glands from *mof; Top2^suo1^/Top2^suo3^* doubly mutant males showed that the X with rescued morphology recruits a normal amount of ISWI despite the absence of Top2 (Figure S5). Thus, Top2 requirement for compensated chromatin modeling appears to be independent of ISWI localization.

## Discussion

### Top2 is required for chromatin organization within prophase nuclei of primary spermatocytes

We have shown that mutations in *Top2* affect chromatin organization within the primary spermatocyte nuclei. During prophase I, wild type spermatocytes exhibit 3 main distinct chromatin clusters, which correspond to the major *Drosophila* bivalents (X-Y, 2-2 and 3-3); the small fourth chromosome bivalent is either separated from these chromatin masses or associated with the X-Y bivalent [51]–[54]. We found that at early growth stages (S1 and S2) the chromatin distribution within the spermatocyte nuclei of *Top2^suo1^/Df* males was not substantially different from wild type, suggesting that spermatogonial divisions are not severely affected. However, at stages S4 and S5 mutant spermatocytes displayed approximately twice as many chromatin masses as their wild type counterparts. In addition, in most mutant nuclei masses of similar size were closely apposed, suggesting a separation of the homologs within each chromatin territory. In the subsequent stages of spermatocyte growth, the number of chromatin masses in mutant nuclei progressively decreased, so that at prometaphase they displayed 3 compact chromatin clumps like their wild type counterparts.

Chromosome behavior during spermatocyte growth and male meiosis has been investigated in previous studies, which revealed a complex pairing mechanism [53]–[58],[78]. The X and the Y pair through their rDNA regions, while no specific euchromatic or heterochromatic pairing sites have been identified for the major autosomes, which are thus likely to exploit a homology-based pairing mechanism [53], [54], [78]. Tagging of allelic chromosome sites using the GFP-Lac repressor/*lacO* system or fluorescent in situ hybridization (FISH) showed that the major autosomes are tightly paired during the S1 and S2 stages. However, pairing is suddenly lost at the S2/S3 transition; the chromosomes remain then unpaired throughout the rest of meiosis but are included in a common nuclear territory until they condense prior to meiotic division [53], [54], [78]. One open problem is the mechanism underlying homolog co-mingling within the territories. Such co-mingling is unlikely to be the result of a canonical meiotic pairing, as the homologs remain uncondensed throughout prophase. It has been thus postulated that during early prophase the homologs might be held together in a single territory by chromatin entanglements [53], [54]. Our results are consistent with this idea and lead us to hypothesize that Topo II plays an active role in generating the entanglements that mediate homolog association. However, it is equally possible that Topo II is required for some kind of chromatin modifications that are important for homolog conjunction within the territories.

The chromatin organization defects within the prophase nuclei of *Top2^suo1^/Df* spermatocytes are very different from those previously observed in *Cap-H2* and *Cap-D3* mutants. In these mutants the chromatin remains diffuse within the spermatocyte nuclei from stage S4 through S6, indicating that the Cap-H2 and Cap-D3 condensin II subunits are required for the formation of the intranuclear territories that comprise the homologous chromosomes [58]. The chromatin organization defect in *Top2^suo1^/Df* spermatocytes it is also different from that caused by mutations in genes mediating achiasmate homolog pairing in *Drosophila* males (*teflon*, *MNM* and *SNM*). Spermatocytes of mutants in these genes display diffuse and slightly expanded chromatin territories during stages S4–S6; at prometaphase they show up to eight distinct chromatin clumps corresponding to unpaired univalents [54]–[57].

Collectively, the available results suggest that condensins (Cap-H2 and Cap-D3), the proteins required for homolog conjunction (*teflon*, *MNM* and *SNM*) and Top2 play distinct roles in chromatin organization during spermatocyte growth. As previously suggested, condensins are essential for territory formation and appear to function in opposition to homolog conjunction [58]. Top2, Teflon, MNM and SNM are all required for proper territory formation and homolog pairing. Top2 is primarily required for homolog conjunction and correct territory organization during stages S4–S6 of spermatocyte growth, whereas Teflon, MNM and SNM are primarily required for meiotic chromosome pairing during prometaphase and metaphase. However, Top2 might have a redundant role in metaphase chromosome pairing that would be masked by the activity of Teflon, MNM and SNM, which would be able to mediate homolog pairing even when Top2 is reduced. The finding that Top2 mediates an aspect of homologous chromosome pairing in males is intriguing, as this enzyme ensures proper biorientation of achiasmatic homologs in females (Hughes and Hawley; cosubmitted). Thus, despite the profound differences between *Drosophila* male and female meiosis, both types of meiotic divisions share a common Top2-depedent mechanism to facilitate achiasmate chromosome pairing.

### Top2 is required for proper chromosome organization and segregation during *Drosophila* male meiosis

Previous studies have shown that loss of Topo II function results in species-specific meiotic defects. In *top2* mutant cells of *S. cerevisiae*, premeiotic DNA synthesis, recombination and chromosome condensation are not affected but cells arrest at metaphase I and do not undergo the first meiotic division. However, *top2 rad50* double mutants, in which recombination and synaptonemal complex formation are suppressed, perform the first meiotic division but not the second [79], [80]. A similar meiotic phenotype has been observed in *Top2* mutant cells of *S. pombe*, which exhibit only a mild defect in the final steps of meiotic chromosome condensation and arrest at metaphase. This arrest is relieved by mutations in *rec7* that strongly reduce recombination [81]. Thus, in both budding and fission yeast, Topo II has little or no role in chromosome condensation but its activity is required for segregation of recombinant chromosomes at meiosis I and, at least in *S. cerevisiae*, for sister chromatid separation at meiosis II.

Studies on meiotic cells from mouse and Chinese hamster injected with Topo II inhibitors did not reveal gross defects in chromosome condensation at the doses used in the experiments. However, the inhibitors induced a substantial meiotic delay and resulted in anaphase bridges and lagging chromosomes at both the first and the second meiotic anaphase [82]–[85]. It has been also suggested that the defect in homolog separation at meiosis I was due to a primary defect in chiasmata resolution [82]. In contrast, studies on mouse pachytene spermatocyte cultured in vitro showed that treatments with the Topo II inhibitors ICRF-193 and teniposide cause drastic defects in chromosome condensation. In teniposide-treated spermatocytes, both chromatin condensation and sister chromatid individualization were strongly affected. The effects of ICRF-193 were milder and some chromosomes managed to condense reaching a diplotene-like configuration [86].

We showed that weak *Drosophila Top2* mutants (*Top2^suo1^/Top2^suo1^* and *Top2^suo2^/Df*) with virtually no defects in brain cell mitoses exhibit strong defects in chromosome segregation during both meiotic divisions of males (this report and ref [46]). In addition, we have shown that in *Top2^suo1^/Df* and *Top2^suo1^/Top2^suo3^* testes all meiotic divisions exhibit severe defects in chromosome structure and segregation. In most cells, the chromosomes formed amorphous metaphase I masses where the sister chromatids were no longer discernible. In addition, these chromatin masses often emanated protrusions that are likely to correspond to stretched pericentric regions. Despite the strong defect in chromosome structure, *Drosophila* spermatocytes did not arrest at metaphase like yeast cells [79] or mouse spermatocytes treated with teniposide [86]. This finding is consistent with the limited delay caused by the spindle checkpoint in *Drosophila* male meiosis and by the inability of this checkpoint to prevent spermatid formation and differentiation [87], [88]. Given that *Drosophila* male meiosis is achiasmatic, our observations strongly suggest that the aberrant meiotic chromosome segregation observed in *Top2* mutant males is the consequence of a primary defect in chromatin folding within the chromosomes. As shown in Figure 3, this defect is particularly evident when the chromosomes are pulled away by the meiotic spindle. In the anaphase-like figures of *Top2* mutants, the chromatids are not individualized and the spindle poles are connected by an irregular network of chromatin threads. This suggests that the chromatin fibers of both the homologous and the heterologous chromosomes as well as those of the sister chromatids remain trapped by multiple entanglements, which prevent correct chromosome segregation during both meiotic divisions.

The observation that Top2 is required for homolog conjunction during early meiotic prophase and then for correct chromosome segregation during anaphase is intriguing. We favor the hypothesis that Top2 has two independent activities, one required for catenation of the homologs within each chromosome territory and one required for proper chromatin folding within the metaphase chromosomes. However we cannot exclude the possibility that Top 2 activity during early prophase results in aberrant chromatin configurations that interfere with the chromatin folding processes leading to proper chromosome assembly.

Frequent anaphase bridges have been also observed in spermatocytes from mutants in the *Cap-H2* and *Cap-D3* genes. However, the morphology of the meiotic chromosomes of these mutants is not severely disrupted as occurs in *Top2* mutants. Judging from the published micrographs, the chromatids of *Cap-H2* and *Cap-D3* mutants are clearly individualized and their appearance is not very different from that of their wild type counterparts [58]. Our data do not provide an explanation of why the meiotic chromosomes of *Drosophila* males are much more sensitive to Top2 depletion than brain chromosomes. We can only envisage that the two types of chromosomes contain different proteins and/or different numbers of high affinity Top2 binding sites. If these binding sites were more frequent in meiotic than in mitotic chromosomes, then a ∼50% reduction of the Top2 protein would be sufficient to disrupt meiotic chromosome organization in males, but would have only a limited effect on mitotic chromosomes of larval brains.

A meiotic phenotype reminiscent of that seen in Top2 mutant males has been observed after RNAi-mediated depletion of Top2 in females (Hughes and Hawley; cosubmitted). In wild type female meiosis, the heterochromatic regions of the homologous chromosomes remain connected during prometaphase I by chromatin threads that ensure proper biorientation of achiasmatic homologues; these homologous connections are then resolved at later stages of meiosis allowing chromosome segregation. In Top2- depleted oocytes, heterochromatic regions of chromosomes usually fail to separate during prometaphase and metaphase I, and are often stretched into long protrusions with centromeres at their tips (Hughes and Hawley and references therein). These findings indicate that Top2 is required for resolution of the DNA entanglements that normally connect homologous heterochromatic regions during female meiosis and suggest that the pulling forces exerted by the spindle generate chromatin protrusions. However, the meiotic phenotypes elicited by Top2 depletion in males and females are similar but not identical. While in female meiosis only the heterochromatic regions appear to be affected, in male meiosis both euchromatin and heterochromatin are affected. A higher sensitivity of heterochromatin to Top2 depletion is consistent with our observations on *Top2^suo1^/Df* and *Top2^suo1^/Top2^suo3^* mitotic cells, which exhibit chromosome breaks that preferentially involve the 3L and the Y heterochromatin.

### *Drosophila* Top2 is required for maintenance of chromosome integrity

We have shown that relatively weak mutant combinations of *Top2* alleles (*Top2^suo1^/Df* and *Top2^suo1^/Top2^suo3^*) only exhibit chromosome aberrations (CABs), most of which involve specific regions of the Y and third chromosome heterochromatin. Severe RNAi-mediated Top2 depletion results in extensive chromosome breakage involving all chromosome regions with a preference for heterochromatin. Previous studies with pharmacological inhibitors of Topo II have also shown that treatments with these drugs cause CABs, but it is currently unclear to which extent these drugs directly induce DNA lesions, cause DNA damage via Topo II inhibition, or affect DNA stability through other off target effects [89]. However, CABs have been also observed in *Top2* mutants of budding and fission yeasts [18], [20],[21],[90] and in vertebrate cells depleted of Topo II by RNAi [17], [24], [25], [32], [91]. In both yeast and vertebrate systems, most CABs induced by Topo II deficiency are thought do be produced by breakage of the anaphase chromatin bridges generated by failure to decatenate sister chromatids [17], [25], [32], [90], [91].

We have shown that a relatively modest reduction of the Top2 level results in many isochromatid breaks and chromosome exchanges (translocations and dicentric chromosomes) that primarily involve 4 regions of the entirely heterochromatic Y chromosome (regions h1-2; h4-6, h19-21 and h24-25) and a specific region of the 3L heterochromatin (region h47). To the best of our knowledge, previous studies did not detect site-specific chromosome aberrations after inhibition of Topo II function. What is then the mechanism underlying the chromosome damage specificity in weak *Top2 Drosophila* mutants? Two observations help answering this question. First, in mutant brain cells not treated with colchicine, 37% of the anaphases displayed chromatin bridges or lagging chromosome fragments generated by severing of the bridges. Second, most CABs observed in colchicine-treated cells were “incomplete” chromosome type aberrations (i.e involving both sister chromatids). Namely, they consisted in broken centric chromosomes not accompanied by a corresponding acentric fragment, in normal chromosome complements with an additional acentric fragment, in Y-3 translocations lacking the reciprocal element, or Y-3 dicentric chromosomes lacking the acentric fragment. As illustrated in Figure 5, these aberrations are likely to be the consequence of chromosome breaks generated during the anaphase of the previous cell cycle. We propose that these breaks preferentially occur in chromosomal sites whose stability is particularly dependent on Top2-mediated DNA decatenation. In Top2 deficient cells, these sites would not be properly untangled and would break when the sister chromatids are pulled apart by the mitotic spindle. It has been shown that a prominent Top2 cleavage target is the 359 bp *Drosophila* satellite DNA, which is mainly found in the centric heterochromatin of the X chromosome [92]. We examined the extant maps of satellite DNA and transposable element distribution along *Drosophila* heterochromatin [93]–[95] but did not find any sequence that uniquely maps to the Top2-sensitive regions. Thus these regions might share an as yet unidentified DNA or might correspond to junctions between different DNA sequences (e. g. satellite-satellite; satellite transposon, or transposon-transposon).

*Top2* RNAi brain cells contain very small amounts of Top2 and exhibit only a few divisions, most of which are hyperploid or polyploid. The few scorable diploid figures almost invariably displayed incomplete aberrations involving the Y or third chromosome heterochromatin, often accompanied by breaks in other chromosomes. We could not assess the presence of incomplete aberrations in polyploid metaphases, most of which displayed many apparent breaks of the centric heterochromatin of the major autosomes. These discontinuities in chromosome structure could be either due to drastic failures of heterochromatin condensation or to real breaks generated by the rupture of chromatin bridges during anaphase. Our results do not permit us to discriminate between these possibilities, but we favor the first.

### The role of Top 2 in the control of larval brain cell cycle

Our observations on different *Top2* mutant combinations and *Top2* RNAi cells revealed different and apparently contradictory effects on cell cycle progression. In *Top2^suo1^/Df* brains that exhibit a ∼60% reduction in the wild type Top2 level, the MI was comparable to that of wild type controls, but mutant brains displayed an increase in the frequency of anaphases. These data are consistent with previous studies on *Drosophila* S2 cells showing that RNAi-mediated depletion of Top2 does not affect the MI and causes only a small increase in the anaphase frequency [40], [42]. The MI was not substantially affected also in DT40 and human cells depleted of both Topo II alpha and Topo II beta [24], [25], [32].

In contrast, in *Top2* RNAi brains and *Top^suo3^/Df* brains the MI was reduced by one and two orders of magnitude, respectively. *Top2* RNAi brains also displayed many aneuploid and polyploid cells. Polyploidy has been also observed in chicken and human cells lacking Topo II activity, and was attributed either to defects in cytokinesis or to a reentry into interphase following a mitotic arrest (restitution) [24], [25]. The low MI and the extensive chromosome damage in RNAi brains did not allow us to reliably pinpoint the mechanism of polyploid cells formation. Polyploidy in *Drosophila* brains can be generated by either restitution or cytokinesis failure (see for example refs [47] and [63]). It is thus possible that the polyploid cells of *Top2* RNAi brains were generated through both mechanisms.

The observations on weak *Top2* mutants (*Top2^suo1^/Df* and *Top2^suo1^/Top2^suo3^*; this study) and *Top2* RNAi S2 cells [40], [42] strongly suggest that *Drosophila* does not have a decatenation checkpoint that arrests cell cycle in response to loss of Top2 function (see Introduction). This conclusion agrees with recent data indicating that Topo II depletion and the resulting excess of DNA catenation does not trigger a G2 arrest in vertebrate cells [24], [25], [38], [96]. However, a catenation-independent but Topo II-dependent checkpoint is activated by interruptions of the decatenation process caused by catalytically inactive forms of Topo II [22], [37], [96].

We found that in *Top2^suo3^/Df* and *Top2* RNAi brains the MI is drastically reduced, indicating that cells are blocked in interphase. We also showed that this block is not relieved by mutations in either *mei-41* (ATR) or *tefu* (ATM). Because these kinases are involved in the signaling pathways that mediate most cell cycle checkpoints [64], [65], and because ATR has been previously implicated in the decatenation checkpoint [35], we believe that the interphase block observed in the nearly complete absence of Top2 is not due to the activation of a checkpoint. We instead believe that this block could depend on the failure to remove supercoils during DNA replication, which would cause extensive DNA damage and make the cell unable to sustain cell cycle progression.

### Top2 is required for chromatin organization of the dosage-compensated X chromosome

We first reported that mutations in *Top2* cause a specific alteration of the X chromosome morphology in male polytene chromosomes (Bonaccorsi et al., 50^th^ *Drosophila* Research Conference; abstract 350b, 2009). This observation was confirmed and extended by Hohl and coworkers [45], who showed that in polytene nuclei of *Top2* mutants the X chromosome retains the ability to recruit the MSL dosage compensation complex. In agreement with this study, we found that the bloated X chromosomes of *Top2* mutants are decorated by anti-Mle, anti-Msl3 and anti-Mof antibodies. However, we have been unable to assess whether the staining intensity is the same as that of a normally condensed wild type X. Thus, it is quite possible that a reduction in Top2 expression partially affects the association of the MSL complex with the male X chromosome as recently suggested [97]. Regardless of the role of Top2 in recruitment and/or stabilization of the MSL complex, the observation that loss or inhibition of Top2 activity specifically disrupts the X chromosome morphology in males is fully consistent with the ChIP/Mass Spec experiments indicating that Top2 is the major MSL interactor [98] and with the co-IP assays showing that Top2 interacts with MSL through its Mle component [97].

Previous studies have shown that the X chromosome of polytene nuclei from *Top2* mutants is decorated by antibodies against histone H4 acetylated at lysine 16 (H4K16ac). This post-translational modification is mediated by the Mof histone acetyltransferase, whose association with the X chromosome depends on Mle; a mutation in *mle* or blocking H4k16 acetylation rescues the X chromosome condensation defects caused by mutations in *ISWI* [76]. We found that the X chromosome phenotype elicited by mutations in *Top2* is rescued in *mof*; *Top2* double mutants, and that both the bloated X of *Top2* mutants and the reconstituted X of *mof; Top2* double mutants normally recruit the ISWI protein. These results suggest that loss of Top2 does not affect condensation of the dosage compensated chromatin by inhibiting ISWI recruitment. However, in the absence of Mof-mediated H4k16 acetylation the chromatin compaction functions of Top2 and ISWI are both dispensable. The genetic interaction between *Top2* and *mof* is consistent with the previously shown physical and functional interactions between topoisomerase II and histone deacetylases (HDACs) [2], and with the synergistic cytotoxic effects caused by simultaneous inhibition of HDAC and Topo II [99], [100].

### Conclusions

We have shown that meiotic chromosomes are extremely sensitive to Top2 depletion and exhibit drastic defects in chromosome morphology even in weak *Top2* mutants. Top2 downregulation in brain mitotic cells by either mutations or in vivo RNAi produced different cytological phenotypes. Moderate Top2 depletion (*Top2^suo1^/Df*) did not affect chromosome structure, and produced site-specific chromosome aberrations generated by the rupture of anaphase bridges. Severe Top2 depletion (*Top2* RNAi) strongly reduced the MI, and induced heterochromatin undercondensation, extensive chromosome breakage, aneuploidy and polyploidy. Finally, complete (or nearly complete) Top2 deficiency (*Top2^suo3^/Df*) caused an interphase block and disrupted chromatid individualization in the rare diving cells. These phenotypes indicate that *Drosophila* chromosomes are exquisitely sensitive to the residual level of Top2 in the cell. In addition, they recapitulate most, if not all, phenotypes previously observed in vertebrate cells exposed to Topo II inhibitors or RNAi against Topo II (see above). Thus, our results suggest that the previously observed discrepancies in vertebrate chromosome phenotypes elicited by Topo II downregulation might depend on the type of chromosomes examined (e.g. mitotic *vs* meiotic), slight differences in Topo II activity, or both.

## Materials and Methods

### Fly stocks and genetics

*Top2^suo1^* and *Top2^suo2^* mutant alleles were previously isolated by a cytological screen of a collection of male sterile mutants induced by EMS in C. Zuker laboratory [46], [101]. *Top2^suo3^* was isolated from a collection of about 1,500 lines carrying lethal mutations on chromosome 2, arisen in the Zucker collection of viable mutants [101]. All mutations were kept in stock over the second chromosome balancer *CyOTbA*, bearing the *Tubby^1^ (Tb^1^)* dominant transgene [102]. *Df(2L)Exel9043* was obtained from the Bloomington Drosophila Stock Center. *mei-41^29D^* [103] was kept in stock over an *FM7-GFP* balancer. The *mei-41^29D^; Top2^suo1^/Top2^suo3^* and *mei-41^29D^; Top2^suo3^/Df(2L)Exel9043* double mutants were obtained by crossing *mei-41^29D^/FM7-GFP; Top2^suo3^/CyOTbA* females to *FM7-GFP/Y; Top2^suo1^/CyOTbA* and to *FM7-GFP/Y; Df(2L)Exel9043/CyOTbA* males, respectively. Male larvae carrying both mutations were identified based on their non-GFP non-*Tb* phenotype. *tefu^3^* (or *atm^3^*, a gift of S. D. Campbell; ref [104]) was kept in stock over the *TM6C* balancer carrying the *Stubble (Sb)* and *Tb* dominant markers. The *Top2^suo1^/Top2^suo3^; tefu^3^/tefu^3^* double mutant was obtained by crossing *Top2^suo1^/CyOGFP; tefu^3^/TM6C* females to *Top2^suo3/^CyOGFP; tefu^3^/TM6C* males. Doubly mutant larvae were identified on the basis of their non-GFP, non-*Tb* phenotype. The *mof^1^* mutant stock was kindly provided by J. Lucchesi. To construct *mof*; *Top2* double mutants we crossed *mof^1^/FM7-GFP*; *Top2^suo1^/CyOTbA* females to *FMT-GFP/Y; Top2^suo3^/CyOTbA* males; non-GFP and non-*Tb* male larvae were then selected for cytological examination of polytene chromosomes. *Iswi* mutant larvae were generated by crossing *y w; Iswi^2^ sp; +/T(2;3)B3 CyO, TM6B* females to *Iswi^1^ Bc*/*SM5, Cy sp* males and recognized for the *Bc* non-*Tb* phenotype (*Iswi* mutant stocks are a gift of D. Corona; see ref [76]).

For in vivo RNAi-experiments, flies carrying a *Top2* RNAi construct (line 4570; VDRC collection) were crossed to males carrying the Actin-Gal4 driver. The Oregon R laboratory strain was used as wild type control. All the stocks were maintained at 25°C on a standard medium. For markers, balancers and special chromosomes details see FlyBase (http://www.flybase.org).

### Western blotting

Extract preparation and Western blotting were performed according to ref. [105] using a rabbit antiserum directed against aa 534–950 of Top2 (gift of P. Fisher; see ref [48]) diluted 1∶2,500. The anti-alpha tubulin (SIGMA) and the anti-lamin (Dm0, Hybridoma Bank) antibodies used for loading control were diluted 1∶20,000 and 1∶2,500, respectively. Bands were detected with Chemi Doc XRS+ (BIORAD laboratories), and densitometric analysis was performed using the Image Lab 4.0.1 software.

### Mitotic and meiotic chromosome cytology

To analyze the morphology and the integrity of metaphase chromosomes, brains from third-instar larvae were dissected in saline (NaCl 0.7%). After incubation for 1 h with colchicine (10^−5^ M in saline), brains were treated for 8 min with hypotonic solution (0.5% Na Citrate), squashed in 45% acetic acid under a 20×20 mm coverslip, and immediately frozen in liquid nitrogen. To analyze anaphases and assess mitotic parameters, larval brains were disssected in saline, directly squashed without colchicine and hypothonic pretreatment and immediately frozen. The mitotic index (MI) was calculated by determining the average number of mitotic figures per optic field as described previously [47]. To visualize meiotic chromosome morphology (Figure 3C), pupal testes isolated in saline were squashed in 45% acetic acid and frozen in liquid nitrogen. For polytene chromosome analysis, salivary glands were dissected in saline, squashed in 45% acetic acid and then frozen in liquid nitrogen. After removal of the coverslip, slides were air dried and mounted in Vectashield H-200 (Vector Laboratories) containing the DNA dye DAPI.

### Immunostaining

To analyze nuclear organization of primary spermatocytes larval and pupal testes were fixed according to ref [52]. To analyze mitosis, brains from third instar larvae were dissected and fixed according to ref [106]. After several rinses in phosphate-buffered saline (PBS) slides were incubated overnight at 4°C with a monoclonal anti-alpha tubulin antibody (Sigma Aldrich) diluted 1∶1,000 in PBS and either a rabbit anti-DSpd-2 (1∶5,000; ref [59]) or a rabbit anti-phosphorylated histone H3 (1∶1000 Millipore), also diluted in PBS. Primary antibodies were detected by 1 hour incubation at room temperature with FITC-conjugated anti-mouse IgG (1∶20; Jackson Laboratories) and CY3-conjugated anti-rabbit IgG (1∶300; Invitrogen), both diluted in PBS. For anti-Top2 immunostaining, larval brains fixed according to ref [107] were incubated overnight with a rabbit anti-Top2 antibody (gift of Donna J. Ardnt-Jovin; ref [49]) diluted 1∶100 in PBS. After rinsing in PBS, slides were incubated for 1 hour at room temperature with a CY3-conjugated anti-rabbit IgG (1∶300).

For polytene chromosome immunostaining, salivary glands were dissected in saline, fixed as described in ref. [108] and incubated overnight with either of the following polyclonal antibodies diluted in PBS: rabbit anti-ISWI (1: 100; gift of D. Corona) and rabbit anti-Mof, (1: 100; gift of J. Lucchesi). CY3-conjugated anti-rabbit IgG (1: 300; Invitrogen) used as secondary antibodies. Immunostained preparations were mounted in Vectashield H-200 (Vector Laboratories) with DAPI.

### Microscopy

All cytological preparations were examined with a Zeiss Axioplan fluorescence microscope, equipped with a cooled charged–coupled device (CCD camera; Photometrics CoolSnap HQ). Grayscale images were collected separately, pseudocolored and merged.

## Supporting Information

Figure S1 **Examples of anaphases observed in** ***Top2^suo1^/Df*** **brains.** The lagging acentric fragments comprise either a pair of banded Y chromosome sister chromatids (arrows) or two paired euchromatic elements (arrowheads), which are probably 3L arms. Heterologous fragments comprising a Y chromatid and a euchromatic element were never observed.
(JPG)

Figure S2 **Examples of aberrations generated by chromosome breaks that occurred during the anaphase of the previous cell cycle.** Panels (3–4) and (3–6) and the corresponding diagrams show Y-3 dicentric chromosomes (asterisks) and acentric fragments (arrows) generated by chromosome breaks that occurred in the anaphase of the previous cell cycle (see text and Figs. 4 and 5 for detailed explanation). Panels C1 and C2 show metaphases with a complex pattern of aberrations, which are also likely to result from breaks produced by rupture of anaphase bridges between entangled sister chromatids. Arrowheads and arrows point to centric and acentric chromosome fragments, respectively; asterisks indicate Y-autosome chromosome exchanges. Scale Bar, 5 µm.
(JPG)

Figure S3 ***Top2*** **RNAi brains contain more residual Top 2 than** ***Top2^suo3^/Df*** **brains.** The image shown was obtained with a longer exposure of the same blot of Fig. 1A. Note the weak Top2 band in the RNAi lane.
(JPG)

Figure S4 **Examples of polytene chromosomes from** ***Top2*** **mutant and** ***Top2*** **RNAi males.** Note that in *Top2^suo3^/Df* and *Top2* RNAi males the X chromosomes no longer exhibit their typical banding pattern.
(JPG)

Figure S5 **Iswi binds the X chromosome of male polytene nuclei from** ***Top2*** **mutant males.** Note that Iswi decorates both the poorly condensed X chromosome of *Top2^suo1^/Top2^suo3^* males and the normally condensed X from *mof*; *Top2^suo1^/Top2^suo3^* doubly mutant males; wt, wild type.
(JPG)
