# Supplementary figures and images for: The Analysis of Mutant Alleles of Different Strength Reveals Multiple Functions of Topoisomerase 2 in Regulation of Drosophila Chromosome Structure
Source: PLoS Genet. 2014 Oct 23;10(10):e1004739. doi: 10.1371/journal.pgen.1004739 (PMC4207652; doi:10.1371/journal.pgen.1004739)

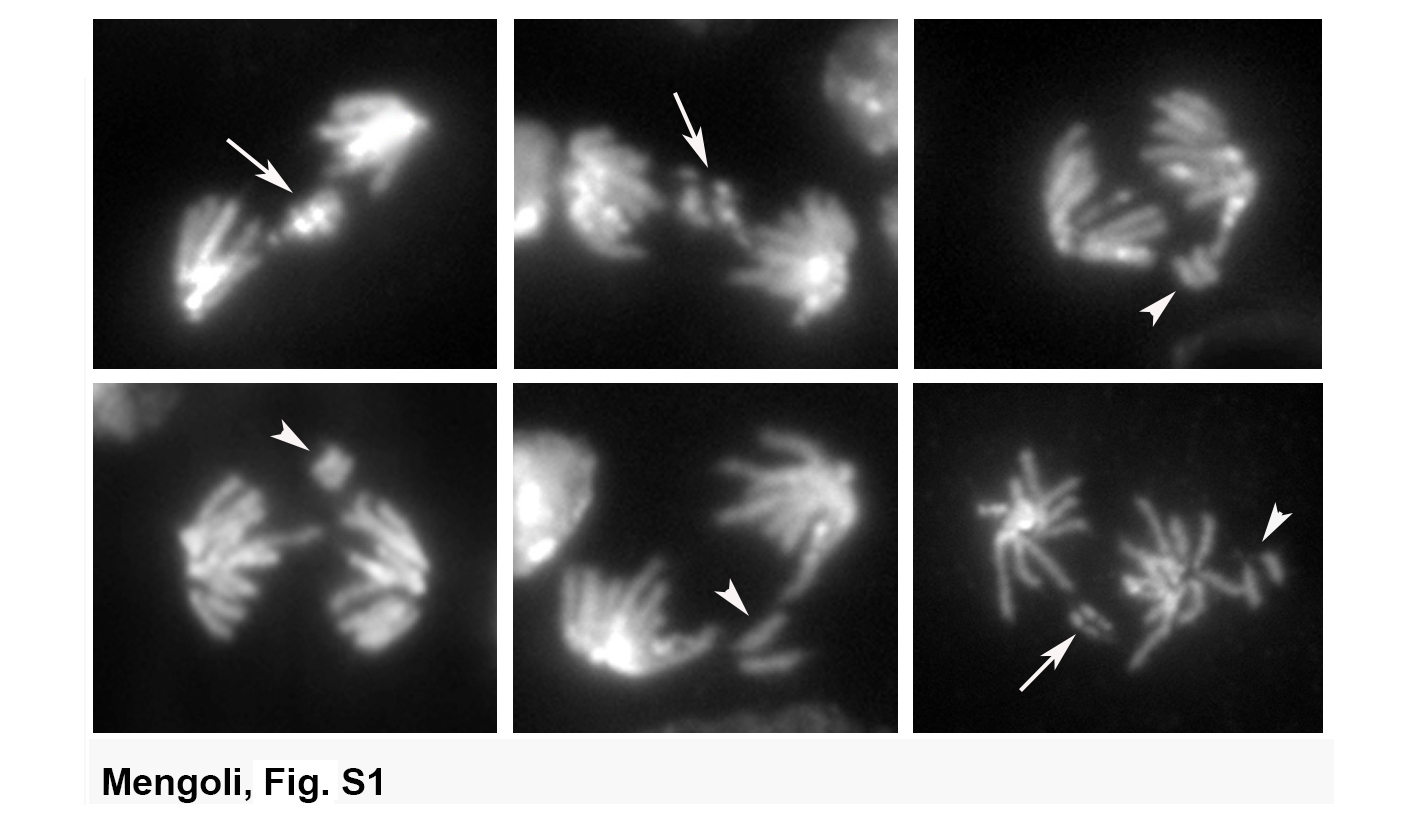

Supplement: Figure S1 — Examples of anaphases observed in Top2suo1/Df brains. The lagging acentric fragments comprise either a pair of banded Y chromosome sister chromatids (arrows) or two paired euchromatic elements (arrowheads), which are probably 3L arms. Heterologous fragments comprising a Y chromatid and a euchromatic element were never observed. (JPG) [file pgen.1004739.s001.jpg]

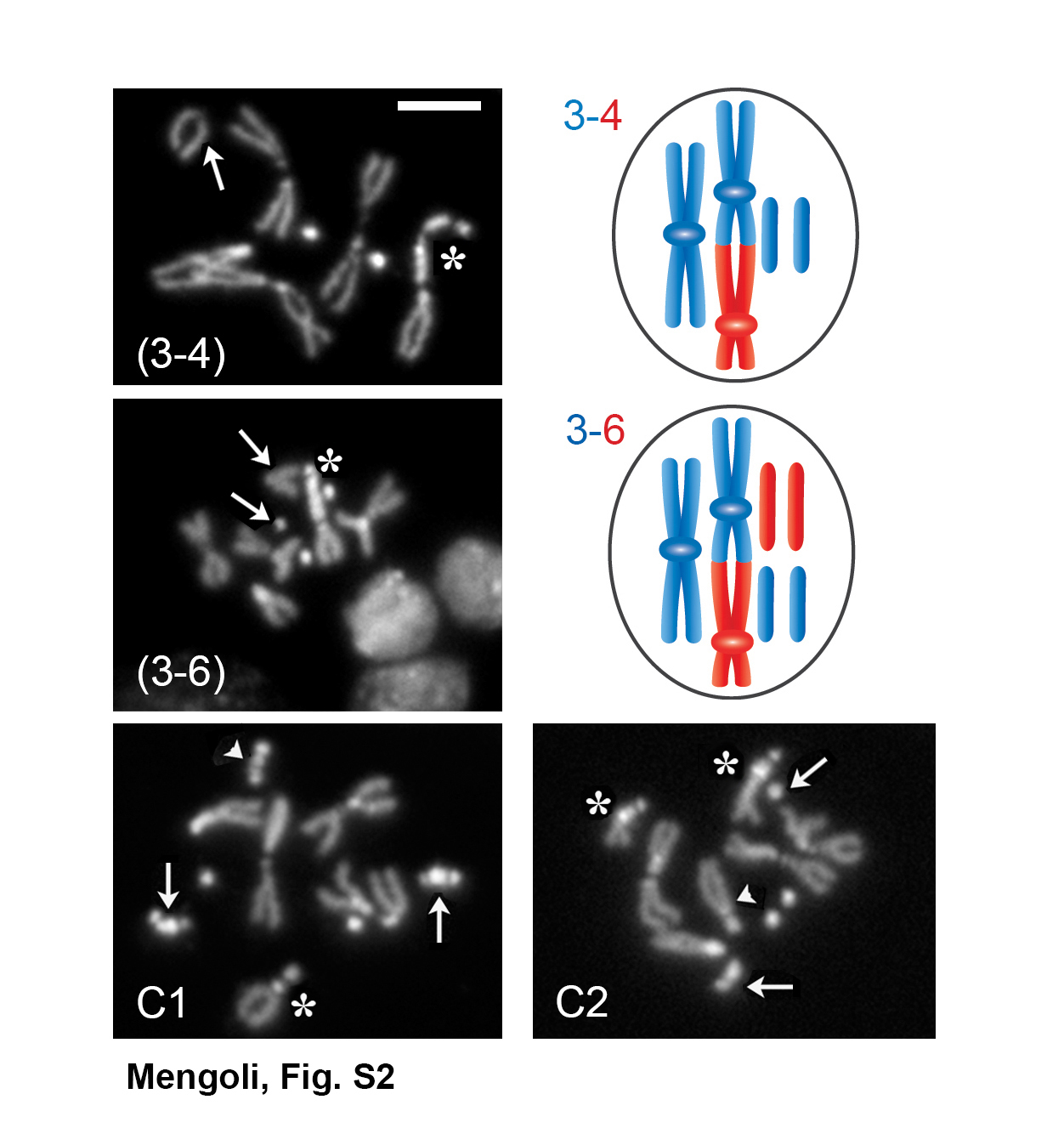

Supplement: Figure S2 — Examples of aberrations generated by chromosome breaks that occurred during the anaphase of the previous cell cycle. Panels (3–4) and (3–6) and the corresponding diagrams show Y-3 dicentric chromosomes (asterisks) and acentric fragments (arrows) generated by chromosome breaks that occurred in the anaphase of the previous cell cycle (see text and Figs. 4 and 5 for detailed explanation). Panels C1 and C2 show metaphases with a complex pattern of aberrations, which are also likely to result from breaks produced by rupture of anaphase bridges between entangled sister chromatids. Arrowheads and arrows point to centric and acentric chromosome fragments, respectively; asterisks indicate Y-autosome chromosome exchanges. Scale Bar, 5 µm. (JPG) [file pgen.1004739.s002.jpg]

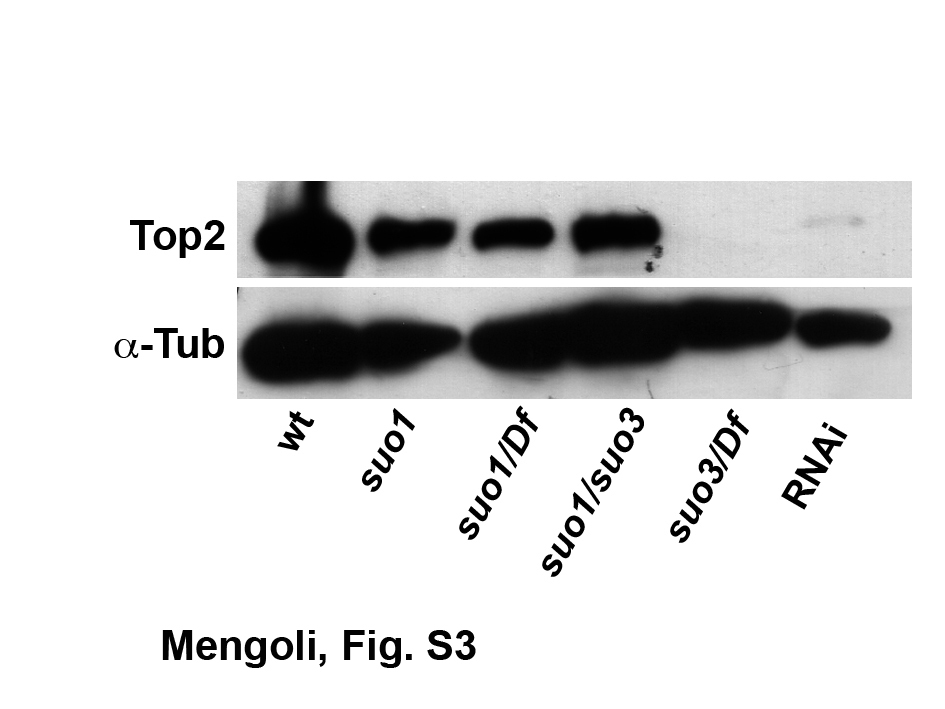

Supplement: Figure S3 — Top2 RNAi brains contain more residual Top 2 than Top2suo3/Df brains. The image shown was obtained with a longer exposure of the same blot of Fig. 1A. Note the weak Top2 band in the RNAi lane. (JPG) [file pgen.1004739.s003.jpg]

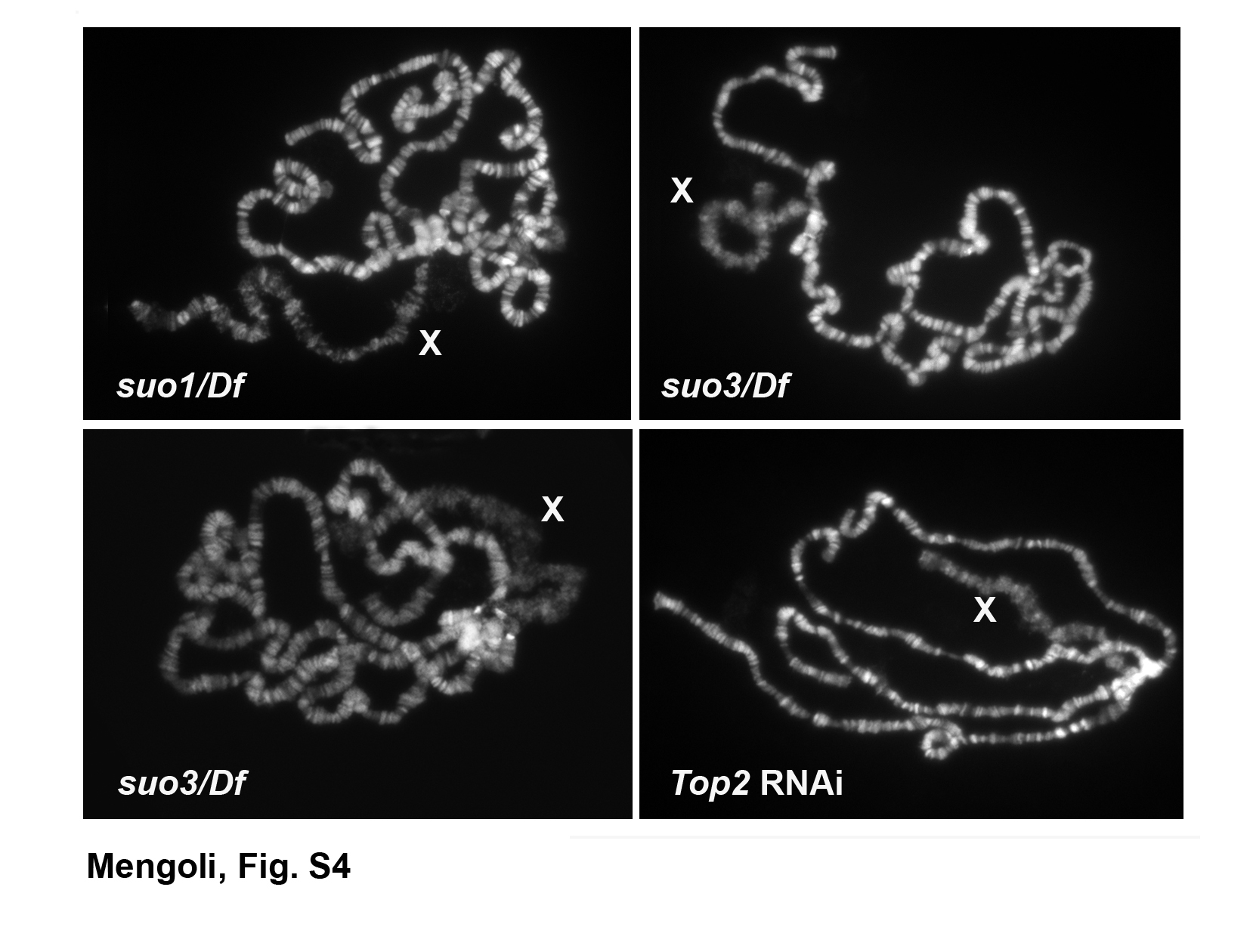

Supplement: Figure S4 — Examples of polytene chromosomes from Top2 mutant and Top2 RNAi males. Note that in Top2suo3/Df and Top2 RNAi males the X chromosomes no longer exhibit their typical banding pattern. (JPG) [file pgen.1004739.s004.jpg]

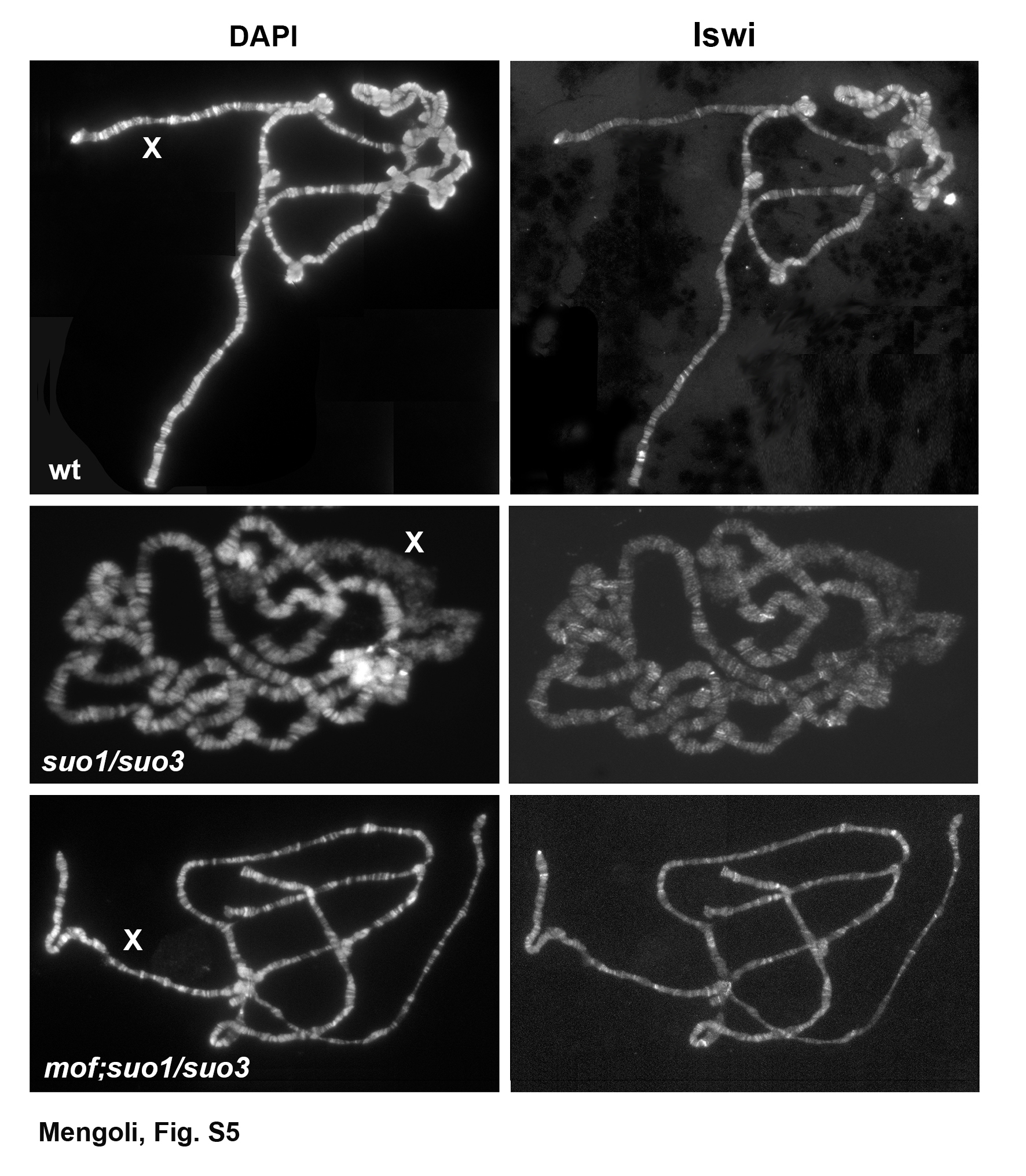

Supplement: Figure S5 — Iswi binds the X chromosome of male polytene nuclei from Top2 mutant males. Note that Iswi decorates both the poorly condensed X chromosome of Top2suo1/Top2suo3 males and the normally condensed X from mof; Top2suo1/Top2suo3 doubly mutant males; wt, wild type. (JPG) [file pgen.1004739.s005.jpg]
